# The platform business model selection of online ride-hailing giants based on the aggregation model

**DOI:** 10.1038/s41598-024-58984-x

**Published:** 2024-04-13

**Authors:** Xueyu Zhu, Min Guo, Jinhong Li

**Affiliations:** 1 School of Computer Science and Technology, North University of China, Taiyuan, 030000 China; 2 College of Economic and Management, North University of China, Taiyuan, 030000 China

**Keywords:** Pure self-management model, Pure aggregation business model, Self-support + aggregation business model, Service preference, Sharing economy, Engineering, Mathematics and computing

## Abstract

Pure self-management model, pure aggregation business model and Self-support + aggregation model are three commonly used business modes on ride-hailing platforms. We use an analytical model to study these three business models and give the optimal business model decision of the platform. The research shows that the heterogeneity ratio of drivers, the cost of the platform under the Self-support model, the franchise fee received by the platform under the aggregation model and the dissatisfaction of the original users on the platform play a key role in the selection of the platform’s business model. When the difference between the franchise fee under the aggregation mode and the platform cost under the Self-support mode fails to generate positive feedback on the platform profit, the platform should choose the pure Self-support mode. When riders are more sensitive to the heterogeneity of service quality of the platform and user stickiness can be ensured, the platform should choose the pure aggregation business model. When user stickiness can be guaranteed and the cost of the platform under the self-run model is controllable, the platform should choose the Self-support + aggregation business model.

## Introduction

In recent years, with the rapid development of economic society and online payment technology, the sharing economy has penetrated into all walks of life. The shared travel industry has gradually developed from the initial industry exploration period to the market start-up period to the later stage of rapid development and the focus of competition has gradually entered the compliance war from the initial price war. At the same time, a new platform business model different from the previous online ride-hailing business model, the aggregation platform, has emerged and become a rising star in the industry, which has gradually threatened the leading position of the traditional platform. Ministry of Transport said, according to the statistics of the online car-hailing supervision information interaction system, a total of 574 million orders were received by October 2022, of which the aggregation platform completed 141 million orders, accounting for 24.56%, up 3.56% from July of the same year^1^. The growth rate is impressive.

In the existing environment, the shared travel industry platform is roughly divided into three business models: pure self-operated platform business model, pure aggregation platform business model and self-operated + aggregation platform business model.

Uber, Lyft, Bolt, etc. are the platforms of the pure self-management model, which is the earliest business model of a shared travel platform. Its business model continues the traditional platform business model, builds a bridge for drivers and passengers, and plays the role of an intermediary. Because the platform using the above business model has the characteristics of high platform compliance rate and higher protection of driver’s rights and interests, the willingness of full-time drivers (i.e. qualified drivers) who pay more attention to long-term benefits to join the platform is higher.

The platforms for the pure aggregation business model include Quick Switch-Uber-Lyft-

Lolipop, Gaode Map, etc. The birth of such platforms is in the context of the oligarchic era of the online car-hailing market. In order to squeeze into the shared travel market, and the low market share of small-scale shared travel platforms, the traffic platform gathers numerous small platforms into its own platform. The platform of such business model is supported by its own strong traffic, through its own traffic to establish the connection between passengers and small platforms, and the momentum of development is rapid. The platform of the pure aggregation business model is different from the previous traditional platform business model. As an intermediary, the platform establishes a bridge for the third-party platform and passengers, and has the right to manage the drivers under the third-party platform. However, because most of the converged platforms are small platforms, the platform compliance rate and the protection of the rights and interests of drivers are low. In order to seize the market share, this kind of platform will subsidize the driver’s price, and pay little attention to the driver’s qualification. Therefore, part-time drivers (i.e., unqualified drivers) who pay attention to short-term benefits are more willing to join.

The platform of self-operated + aggregated business model has Didi Chuxing, Baidu Map and so on. With the development of the aggregation platform, such platforms are becoming more and more prosperous. In order to improve the platform’s capacity and seize the market share, the original online car-hailing giant platform had to incorporate some small platforms into its own. The platform of this business model is a platform that has both the above two business models.

Due to the fact that the current shared travel market is in short supply, each platform must first improve its own capacity if it wants to improve its market share. At this time, the platform is crucial to the service quality of the driver, that is, the driver’s heterogeneous sensitivity to this, in order to attract the driver to join their own platform. However, in the process of industry development, many platforms only focus on the needs of passengers to achieve the purpose of winning more consumers, but ignore the needs of drivers to a large extent. For example, according to Internet riders, after receiving passenger complaints, the aggregation platform hastily fined and sealed the platform riders without knowing the situation. The self-operated platform is more humane in the face of the above situation. Generally, the passengers are appeased first, and then choose whether to punish the driver after understanding the facts. On February 24, 2022, the Ministry of Transport held a press conference. Officials said that there was a vacuum in the protection of the rights and interests of online car-hailing drivers^2^. Therefore, the platform to improve the quality of service for drivers is the key to improve the platform capacity of the shared travel platform. Uber pays more attention to the protection of the rights and interests of drivers, and Gaode ignores the protection of the rights and interests of drivers, and Didi is between the two.

The main purpose of this paper is to determine what kind of business model should be chosen by the giant platform in the ride-sharing industry from the perspective of the service quality of the platform to drivers. The main problems to be solved are: (1) What is the optimal reward of shared travel platform riders and the best business model of the platform? (2) Under different business models, what impact will the driver’s preference for platform service quality have on platform profits? (3) Is there an optimal business model for the platform? This paper analyzes the above problems by establishing a model, considering the preference of heterogeneous drivers for the service quality of the platform under different business model platforms, so as to analyze the platform profit and driver’s compensation.

We apply a model to answer the above questions. We believe that after the emergence of the online car-hailing aggregation platform, the online car-hailing platform in the shared travel market can choose a pure self-operation mode, a pure aggregation operation mode, or both. This paper considers the heterogeneous sensitivity of heterogeneous drivers to the platform’s services under different business models adopted by the platform, and analyzes the market results and price settings. In addition, we consider different types of drivers in the extended model, including drivers with different sensitivity to the platform’s service quality and drivers with multi-homing behavior and single-homing behavior.

In terms of theoretical significance, based on the evolutionary game theory, this paper constructs the optimal driver reward model of the online ride-hailing platform under the aggregated business model, considers the driver reward problem under the pure self-operation mode, the pure aggregation business model and the self-operation + aggregation business model, and the platform business model selection problem, which fills the gap in the research of sharing economy and bilateral platform. In terms of practical significance, this paper provides suggestions for the selection of business models for existing shared travel platforms. In particular, giant platforms such as Uber, Quick Switch-Uber-Lyft-Lolipop, Didi, and Autonavi Travel provide advice on how to improve platform capacity from the heterogeneity of drivers.

## Literature review

The online ride-hailing platform is a typical two-sided market, which connects two independent user groups-travel users and riders. These two groups can provide network benefits to each other^3^. The research content of this paper mainly focuses on the influence of the service quality preference of the platform based on the driver’s preference for different business models on the choice of different business models of the platform in the shared travel market.

First of all, for the self-operated business model, scholars’ research mainly focuses on the platform’s pricing based on passenger heterogeneity, platform income distribution and pricing based on passenger heterogeneity and platform price competition. However, there are few studies on the heterogeneity of drivers. Wei et al.’ s network externality based on the two-sided market considers the impact of the heterogeneity of passengers’ waiting time on the platform’s business model decision-making in the monopoly market. The results show that the time-sensitive cost of heterogeneous passengers and the operating cost of the platform’s self-operated vehicles play a key role in the platform’s choice of optimal mode^4^. Benjaafar established a model in which the heterogeneity of consumers’ pay and utility for private transportation interacts with carpooling platforms. They consider the impact of traffic congestion and distinguish the conditions for carpooling as a P2P service or B2C service^5^. Kung et al. proposed a profit maximization model for bilateral platforms considering network externalities. This paper focuses on three different pricing strategies, and puts forward the influence of consumers’ price sensitivity on strategy selection^6^. Aloui and Jebsi studied the optimal pricing problem of private platform and Ramsey bilateral monopoly platform under competition. The research shows that the price strategy depends on the price elasticity of demand and the marginal congestion cost^7^. Bryan and Gans, Luke et al. discussed the travel pricing problem of the platform under a variety of online car-hailing market structures on the basis of considering the cross-network externalities of the bilateral market^8,9^. In terms of the aggregation business model, Li Hao and Xiao Qing constructed the Steinberg game model of the platform and the two service providers, and discussed the pricing problem of the online car-hailing market under the aggregation model^10^. On this basis, Li Hao and Xiao Qing established a duopoly price game model under the condition that consumers pay different attention to the platform and have preference for the aggregation mode, and analyzed the competitive equilibrium and influencing factors of the differentiated aggregation platform^11^. Cao Yu et al. constructed a duopoly platform competition model, and studied the selection of large platform aggregation strategies under different network externalities and user travel costs^12^. Xu et al. studied the pricing and service level decisions of the ride-hailing platform under different channels by establishing a competition model between the aggregation channel and the direct channel^13^. Zhou et al. established a competition model of aggregation channel and direct channel of online ride-hailing platform to study the impact of aggregation platform on the entire online ride-hailing market^14^.

In the past, the platform pricing of the two-sided market either considered the pricing of the network externality for the platform alone or considered the influence of passenger heterogeneity on the platform pricing decision and the influence of the driver on the platform pricing, but did not consider the influence of the heterogeneity of the driver for the platform service on the platform. In the research of the aggregation platform, the research focuses on the competitive pricing based on the single platform or multi-platform of the passenger side. Considering the network externality of the two-sided market, the influence of the heterogeneity of the driver on the choice of the platform’s business model is not taken into account, and the choice of the business model of the online ride-hailing platform and the large traffic platform under the aggregation mode is not involved. Firstly, based on the network externalities of the two-sided market, this paper will study the impact of the heterogeneity of drivers on the business model of the online car-hailing platform. Secondly, this paper focuses on three different business models under the aggregation economy, that is, from the pure self-operation mode, the self-operation plus aggregation mode, and the pure aggregation mode to find the optimal business model of the platform based on the heterogeneity of the driver, the cost of the platform’s self-operation team, the joining fee of the platform under the aggregation mode and the dishonesty rate.

## Basic model

Considering the giant platform of self-operation of online car-hailing under the background of the current aggregation platform and the traffic platform ready to enter the online car-hailing market, how to choose the business model based on the heterogeneous preference of drivers for different business models. There are three business models of online car-hailing platforms. The first is a pure self-operated platform (PSP). Under this business model, the online car-hailing platform has its own fleet and franchised fleet. The second is the pure aggregation platform (PAP). Under this business model, the platform only provides traffic by aggregating small online ride-hailing platforms, and does not provide fleets and drivers. The third is the self-operated + aggregation platform (S + A-P), which acts as the role of the first and second business models at the same time. This paper considers that social vehicles can be allowed to enter the online car-hailing market at the same time. The full-time online car-hailing drivers are divided into cakes, and it is assumed that the drivers have no multi-homing behavior.

This paper mainly studies the optimal business model decision-making problem of online car-hailing platform. We assume that a driver can only make one order at the same time, and the driver cannot refuse when receiving the platform order, and assume that the supply and demand are balanced. Then the total number of orders is the total supply of the platform, assuming that the total supply is equal to the total demand. The driver chooses whether to provide services for the platform. If the service quality provided by the platform to the driver is not in line with their expectations when the driver provides the service, it will generate psychological costs. We call this cost as a satisfaction-sensitive cost, which represents the heterogeneity of the driver. According to the heterogeneity of dissatisfaction sensitivity, the drivers are evenly distributed along the unit line. Without loss of generality, the market is distributed between 0 and 1. We use s, a and s + a as the subscripts of pure proprietary platform, aggregation platform and proprietary + aggregation platform, respectively. The basic model focuses on the influence of the driver. Table 1 summarizes the main symbols in the model.

**Table 1:** Key symbols and descriptions of this article.

Denotational description	
$\gamma$	The sharing coefficient of the driver of the platform’s own fleet under the self-operation mode
$\mathrm{\alpha }$	The sharing coefficient of platform franchise network car drivers under the self-operation mode
$\upbeta$	Under the self-operation mode, the platform’s preference coefficient for the drivers of its own fleet
$\phi$	The order preference coefficient for different platforms in the aggregation mode
$u_{i}$	Utility function when the rider provides service ($i = s,a, + s, + a$)
$f_{i}$	Fixed utility when the driver provides service
$h_{i}$	The reward of the driver when providing service
$a_{i}$	Driver’s dissatisfaction sensitive cost when providing unit service
$P_{j}$	The profit when the platform chooses the j business model, j = PSP, PAP, S + A-P
$s_{j}$	The driver supply when the platform chooses the j business model
$d_{j}$	Passenger demand when the platform chooses j business model
$o_{j}$	Platform pricing when the platform chooses the j business model
$\omega$	Drivers’ preference for platform service quality
$\mu$	The commission coefficient of the polymerization platform to join the small platform

### Pure self-operated platform

Under the pure self-operated platform management mode, the platform has its own fleet and franchised fleet, and the driver can join the platform as a driver of its own fleet. In this case, the vehicle is provided by the platform. You can also become a driver to join the platform, which requires the driver to have his own vehicle, in order to obtain orders and income. The order of the joining platform driver is $\alpha$, and the order of the free team driver is $\gamma$, so the cost paid by the passenger when enjoying the joining driver service is $\frac{{h_{s} }}{\gamma }$, and the cost paid when enjoying the self-employed driver service is $\frac{{h_{s} }}{\alpha }$. On this basis, the platform will send orders to the drivers of its own fleet with a preference coefficient of $\beta$, and the preference coefficient for the drivers of the franchise platform is $1 - \beta$, which can also be defined as the openness of the platform. After receiving the platform dispatch order, the driver serves the order and gets paid after the end of the order. At the same time, the driver will generate a satisfaction sensitive cost $a_{s}$, $\omega$ for the driver’s preference for the service quality of the platform (because the service quality of the platform for the driver under each business model is an important measure for the driver to choose whether to live on the platform beyond the income of the driver, refer to Cheng et al. in the study of the software company’s software for the customer free trial to choose the model of the passenger’s prior belief as an indicator^15^, so this article selects the indicator. Therefore, the driver’s anti-utility function^4^ (that is, the higher the driver’s dissatisfaction rate at this moment means that the driver is less willing to provide services on the platform) is:

1 $$ u_{s} = f_{s} - h_{s} - a{}_{s}\omega $$

**Figure 1:**
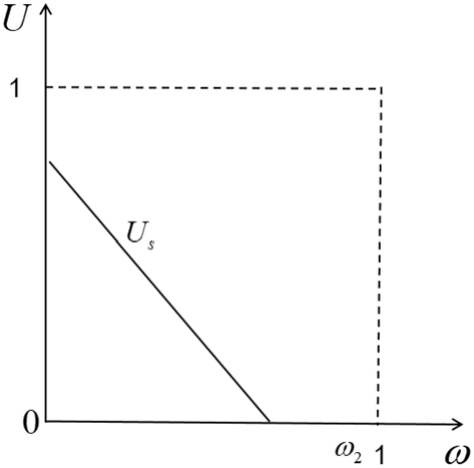
Utility function of driver in pure self-operation mode.

Drivers usually choose the most favorable platform to provide services and maximize their own benefits. As shown in Fig. 1, located at $0 - \omega_{1}$. The drivers between them will choose a pure self-operated platform to provide services, because they can get more benefits in the process of providing services than not providing services. By setting Eq. (1) to 0, we can get the intention of marginal drivers to choose whether to provide services through a pure self-operated platform. The indiscriminate points are:

2 $$ \omega_{1} = \frac{{f_{s} - h_{s} }}{{a_{s} }} $$

In order to ensure that the driver’s supply is non-negative, then the platform of the model should meet the following intrinsic value: $h_{s} \le f_{s} \le h_{s} + a_{s}$, That is, only the driver’s utility is positive, the driver will be willing to provide services.

We assume that a driver can only serve one order, and the supply of the platform can be obtained according to the indifference: $s_{s} = \frac{{f_{s} - h_{s} }}{{a_{s} }}$ (That is, demand is $d_{s} = s_{s} = \frac{{f_{s} - h_{s} }}{{a_{s} }}$), The cost of own fleet is NW, so the platform profit is:

3 $$ P_{psp} = \left[ {\beta \frac{1 - \gamma }{\gamma } + \left( {1 - \beta } \right)\frac{1 - \alpha }{\alpha }} \right]h_{s} \frac{{f_{s} - h_{s} }}{{a_{s} }} - NW $$

By solving the first-order derivative of Eq. (3), the optimal reward of the driver, the optimal pricing of the platform, the optimal supply and the optimal profit can be obtained.

#### Proposition 1

In the pure self-management mode, when the driver’s fixed utility is satisfied $h_{s} \le f_{s} \le h_{s} + a_{s}$, The optimal reward of the driver is $h_{s}^{ * } = \frac{{f_{s} }}{2}$, The optimal supply of the platform is $s_{s}^{ * } = \frac{{f_{s} }}{{2a_{s} }}$, The optimal pricing of the platform is ${\text{o}}_{s}^{ * } = \frac{{f_{s} }}{2\gamma } + \frac{{f_{s} }}{2\alpha }$.

The optimal profit of the platform is $P_{psp}^{ * } = \left[ {\frac{{\beta \left( {1 - \gamma } \right)}}{\gamma } + \frac{{\left( {1 - \alpha } \right)\left( {1 - \beta } \right)}}{\alpha }} \right]\frac{{f_{s}^{2} }}{{4a_{s} }} - NW$. (The proof is in the supplementary information).

**Figure 2:**
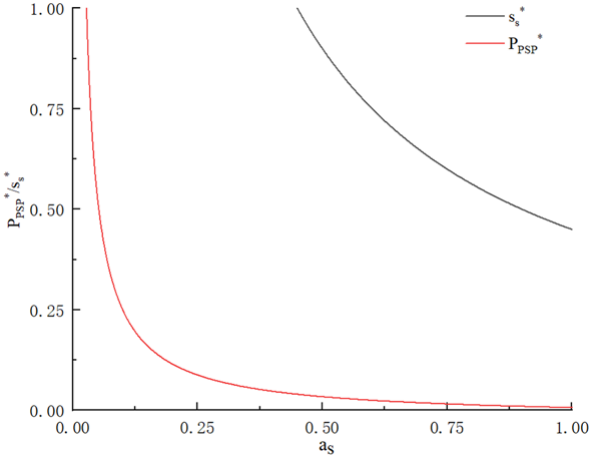
Impact of dissatisfaction on driver supply and platform profit.

According to the optimal results, it can be obtained that in the pure self-operation mode, the optimal reward of the driver increases with the increase of the inherent fixed value of the platform. It shows that the higher the intrinsic fixed value of the platform, the more the drivers will be paid, the more riders will want to join, and it also means that the demand and profit of the platform will increase. With the increase of riders’ dissatisfaction with the platform, the optimal profit and optimal supply of the platform will decrease. If the platform can reduce the driver’s dissatisfaction, it can increase the supply and profit of the platform. The influence of platform openness on profit depends on the sharing coefficient of the platform for its own fleet and franchised fleet. When the sharing coefficient of franchised fleet is greater than the sharing coefficient of its own fleet, the platform profit will increase with the increase of platform openness. When the sharing coefficient of franchised fleet is less than the sharing coefficient of its own fleet, the platform profit will decrease with the increase of platform development. When the platform chooses the pure self-operation mode, it is necessary to improve the openness of the platform and give a higher sharing coefficient to the franchised fleet, which is conducive to improving the capacity and then increasing the profit. (The specific situation is shown in Fig. 2, and the examples are $\alpha = 0.5,\;\beta = 0.6,\;\gamma = 0.3,\;f_{s} = 0.8,\;N = 1,\;W = 0.02$).

### Pure polymerization platform

**Figure 3:**
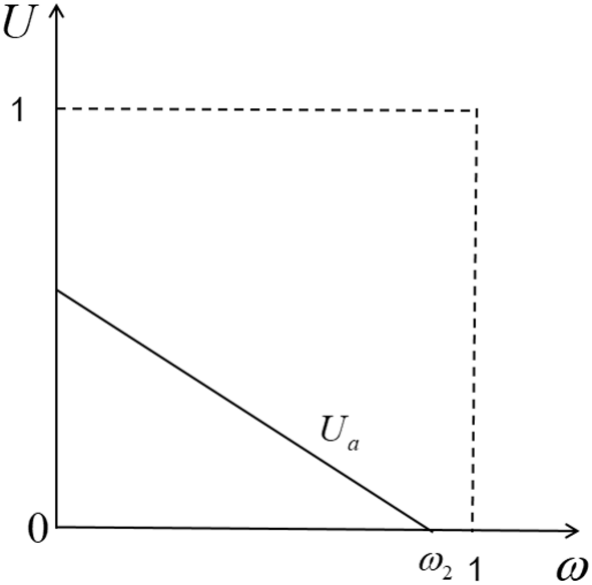
Utility function of driver in pure aggregation mode.

Under the pure aggregation business model, the platform does not provide a fleet. By using its own traffic, many small online car-hailing platforms are aggregated into its own platform. Passengers place orders through the aggregation platform, and the aggregation platform then distributes the order to the small platform as $\phi$ a preference coefficient. Suppose that only two small platforms are added to the large platform, and the commissions on the two platforms are the same, denoted by $\mu$, Since the aggregation platform does not provide a fleet, all drivers are in the franchise mode for the aggregation platform, and the distribution of benefits refers to the pure self-operated platform alliance mode driver allocation mechanism. Therefore, the driver’s counter-utility function under this business model is (Fig. 3):

4 $$ {\text{u}}_{a} = f_{a} - h_{a} - a_{a} \omega $$

Indicates the driver’s service preference to the platform after completing the order under the aggregation mode platform. By setting (4) = 0, we can get the intention degree of the edge driver on whether to choose to provide service through the pure self-owned platform. The undifferentiated point is

5 $$ \omega_{2} = \frac{{f_{a} - h_{a} }}{{a_{a} }} $$

According to the indifference we can get the maximum supply of the platform $s_{a} = \frac{{f_{a} - h_{a} }}{{a_{a} }}$, For the driver’s supply is non-negative, that is $0 \le \omega_{2} \le 1$, Then the platform of this model should meet the following intrinsic value $h_{a} \le f_{a} \le a_{a} + h_{a}$, It shows that the basic utility of the driver is within a threshold. When the utility is lower than the lower bound of this threshold, no driver is willing to provide services under this business model. When the utility is higher than the upper bound of this threshold, there is no platform to enter the market. According to the characteristics of this business model, we can get the profit function:

6 $$ P_{PAP} = \mu \frac{1 - \alpha }{\alpha }{\text{h}}_{a} \left( {\frac{{f_{a} - h_{a} }}{{a_{a} }}\phi + \frac{{f_{a} - h_{a} }}{{a_{a} }}\left( {1 - \phi } \right)} \right) + NIF - NFR $$

Through the first-order derivation of (6), the optimal reward of the driver and the optimal supply, optimal pricing and optimal profit of the platform under this business model can be obtained.

#### Proposition 2

In the pure aggregation platform business model, when the driver’s fixed utility is satisfied ${0} \le f_{a} \le 2a_{a}$, The optimal reward of the driver of the platform is ${\text{h}}_{{\text{a}}}^{ * } = \frac{{f_{a} }}{2}$, The optimal supply of the platform is ${\text{s}}_{a}^{ * } = \frac{{f_{a} }}{{2a_{a} }}$, The optimal pricing of the platform is ${\text{o}}_{a}^{ * } = \frac{{f_{a} }}{2\alpha }$, The optimal profit of the platform is $P_{PAP}^{ * } = \frac{{\mu \left( {1 - \alpha } \right)f_{a}^{2} }}{{4\alpha a_{a} }}$. (The proof is in the supplementary information).

**Figure 4:**
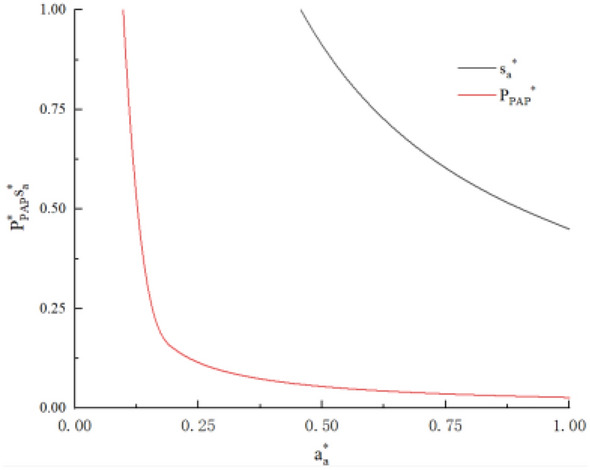
Impact of dissatisfaction on driver supply and platform profit.

According to the results, under the business model of pure aggregation platform, the profit and supply of the platform decrease monotonously with the increase of driver’s dissatisfaction, and decrease monotonously with the increase of fixed utility. In order to have sufficient capacity for the platform, the platform should appropriately increase the proportion of drivers to be divided, or give drivers a certain subsidy. At the same time, it should improve the efficiency of solving complaints about drivers. At the same time, the platform should strengthen the qualification audit for joining the platform and strive to improve the order compliance rate of its own platform, so as to reduce passenger complaints and improve the satisfaction rate of drivers. (As shown in Fig. 4, the examples are the same as above, $f_{a} = 0.9$).

### Self-operated + aggregation platform

Under the business model of self-operated + aggregation platform, the platform acts as both self-operated platform and aggregation platform. As a self-operated platform, it has its own fleet and franchise fleet, and the profit model is the same as the pure self-operated platform. As an aggregation platform, he only has a franchise team, and the profit model is the same as the pure aggregation platform. Therefore, the reverse utility function of the driver under this business model is:

aggregation platform:

7 $$ {\text{u}}_{ + a} = f_{a} - h_{ + a} - a_{a} \omega $$

self-operating platform:

8 $$ {\text{u}}_{ + s} = f_{s} - h_{ + s} - a_{a} \omega $$

**Figure 5:**
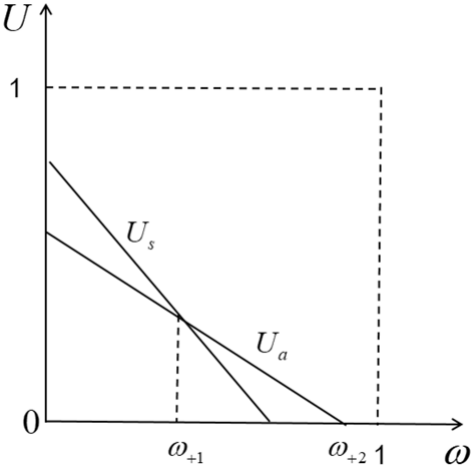
Driver’s utility function under self-operation + aggregation operation mode.

According to Fig. 5, we can see that the utility function divides the driver into three parts. Between $0{-\!\!-}\omega_{ + 1}$ them are drivers who prefer to use pure proprietary platforms for services.

Between $\omega_{ + 1} - \omega_{ + 2}$ is the driver who prefers to use a pure aggregation platform for service. Between $\omega_{ + 2} - 1$ them is the driver who does not use the aggregation platform and does not apply to the self-use platform, that is, the traditional online car-hailing. On the $\omega_{ + 1}$ is the driver who believes that there is no difference between the self-operated platform and the aggregation platform. let $u_{ + a} = u_{ + s}$ and $u_{ + s} = 0$, Two indifference points are obtained.

9 $$ \omega_{ + 1} = \frac{{f_{a} - h_{ + a} - f_{s} - h_{ + s} }}{{a_{a} - a_{s} }} $$

10 $$ \omega_{ + 2} = \frac{{f_{s}^{{}} - h_{ + s} }}{{a_{s} }} $$

According to the indifference point, the supply of the platform can be obtained:

$$ {\text{aggregation}}\;{\text{platform}}:\;s_{ + a} = \omega_{ + 2} - \omega_{ + 1} = \frac{{f_{a} - h_{ + a} }}{{a_{a} }} - \frac{{f_{s} - h_{ + s} - f_{a} + h_{ + a} }}{{a_{s} - a_{a} }} $$

$$ {\text{self - operating}}\;{\text{platform}}:\;s_{ + s} = \omega_{ + 1} = \frac{{f_{s} - h_{ + s} - f_{a} - h_{ + a} }}{{a_{s} - a_{a}^{{}} }} $$

In order to simplify the calculation, reduce the complexity of the calculation, easy to describe. Let $k_{1} = \frac{{\beta \left( {1 - \gamma } \right)}}{\gamma }\frac{{\left( {1 - \alpha } \right)\left( {1 - \beta } \right)}}{\alpha }$, $k_{2} = \frac{{\mu \left( {1 - \alpha } \right)}}{\alpha }$. Let $\theta_{a} = a_{s} - a_{a} ,\;\theta_{a} \ge 0$ representation heterogeneity of drivers’ sensitive cost to service quality satisfaction under the two business models of aggregation platform and self-operated platform. Let $\theta_{f} = f_{a} - f_{s} ,\;\theta_{f} \ge 0\left( {0,\omega_{ + 1} } \right),\;\theta_{f} \le 0\left( {\omega + 1,\omega + 2} \right)$, it indicates the heterogeneity difference of the fixed utility of the driver under the two operating modes of the aggregation platform and the self-operated platform. At this time, the driver supply of the platform can be rewritten as:

11 $$ {\text{Self - operating}}\;{\text{platform:}}\;s_{ + s} = \frac{{\theta_{f} - h_{ + s} + h_{ + a} }}{{\theta_{a} }} $$

12 $$ {\text{Aggregation}}\;{\text{platform:}}\;s_{ + a} = \frac{{f_{a} a_{s} - a_{a} f_{s} + a_{a} h_{ + s} - a_{s} h_{ + a} }}{{a_{a} \theta_{a} }} $$

In order to ensure that the driver supply of the platform is positive, the following conditions should be met: $h_{ + s} - h_{ + a} \le \theta_{f} \le \theta_{f} + h_{ + s} - h_{ + a}$ and $h_{ + a} \le f_{a} \le f_{a} + a_{a}$. In the self-operated + aggregated business model, the benefit function under the platform equilibrium is:

13 $$ P_{s + a} = \beta \frac{1 - \gamma }{\gamma }h_{ + s} s_{ + s} + \frac{{\left( {1 - \alpha } \right)\left( {1 - \beta } \right)}}{\alpha }h_{ + s} s_{ + s} + \frac{{\mu \left( {1 - \alpha } \right)}}{\alpha }h_{ + a} \left[ {s_{ + a} \phi + s_{ + a} \left( {1 - \phi } \right)} \right] - NW_{ + s} $$

The upper profit function includes five aspects, the platform income under the self-operation mode, the cost of the own fleet, the platform income under the aggregation operation mode, the dissatisfaction cost of the platform, and the franchise fee of the small platform. put $k_{1} ,k_{2} ,s_{ + a}$, $s_{ + s}$ into $P_{s + a}$, The profit function can be rewritten as:

14 $$ P_{s + a} = k_{1} h_{ + s} \frac{{\theta_{f} - h_{ + s} + h_{ + a} }}{{\theta {}_{a}}} + k_{2} h_{ + a} \frac{{f_{a} a_{s} - a_{a} f_{s} - a_{s} h_{ + a} + a_{a} h_{ + s} }}{{a_{a} \theta_{a} }} - NW_{ + s} $$

In order to satisfy the profit function is concave function, must satisfy $\frac{{a_{a} }}{{a_{s} }} < \frac{{4k_{1} k_{2} }}{{\left( {k_{1} + k_{2} } \right)^{2} }}\;\frac{{a_{a} }}{{a_{s} }}$ indicates the ratio of heterogeneity difference between drivers who provide services through proprietary platforms and those who provide services through aggregation platforms. Solving $P_{s + a}$ first-order partial derivative to $h_{ + a}$, $h_{ + s}$, we can get

15 $$ h_{ + a}^{ * } = \frac{{\left( {k_{1}^{2} - k_{1} k_{2} } \right)a_{a} \theta_{f} + 2k_{1} k_{2} f_{a} \theta_{a} }}{{4k_{1} k_{2} a_{s} - \left( {k_{1} + k_{2} } \right)^{2} a_{a} }} $$

16 $$ h_{ + s}^{ * } = \frac{{\left( {k_{1}^{2} + k_{1} k_{2} } \right)\left( {f_{a} \theta_{a} - a_{a} \theta_{f} } \right) + 2k_{1} k_{2} \theta_{f} a_{s} }}{{4k_{1} k_{2} a_{s} - \left( {k_{1} + k_{2} } \right)^{2} a_{a} }} $$

Through the optimal income of the driver in the equilibrium state, the optimal supply of the platform can be obtained:

17 $$ s_{ + a}^{ * } = \frac{{k_{1} \left( {k_{1} + k_{2} } \right)a_{a} f_{s} - 2k_{1} k_{2} a_{s} f_{s} }}{{a_{a} \left[ {\left( {k_{1} + k_{2} } \right)^{2} a_{a} - 4k_{1} k_{2} a_{s} } \right]}} $$

18 $$ s_{ + s}^{ * } = \frac{{2k_{1} k_{2} \theta_{f} - \left( {k_{1} k_{2} - k_{2}^{2} } \right)f_{a} }}{{4k_{1} k_{2} a_{s} - \left( {k_{1} + k_{2} } \right)^{2} a_{a} }} $$

Therefore, the optimal profit of the platform is:

19 $$ \begin{aligned} P_{s + a}^{ * } & = \left( {k_{1} + k_{2} } \right)h_{ + s}^{ * } s_{ + s}^{ * } + \left( {k_{3} + k_{4} } \right)h_{ + a}^{ * } s_{ + a}^{ * } - NW_{ + s} \\ & = \frac{\begin{gathered} \left( {7k_{1}^{3} k_{2}^{2} + 2k_{1}^{2} k_{2}^{3} - 2k_{1}^{4} k_{2} + k_{1}^{{}} k_{2}^{4} } \right)f_{a} f_{s} a_{a}^{2} a_{s} + \left( {18k_{1}^{2} k_{2}^{3} + 3k_{1} k_{2}^{4} + 3k_{1}^{3} k_{2}^{2} } \right)f_{a}^{2} a_{a}^{2} a_{s} \hfill \\ + \left( {2k_{1}^{3} k_{2}^{2} - 2k_{1} k_{2}^{4} } \right)f_{a}^{2} a_{s}^{2} a_{a} + \left( {18k_{1}^{4} k_{2} - 9k_{1}^{3} k_{2}^{2} - 2k_{1}^{2} k_{2}^{3} - k_{1} k_{2}^{4} } \right)f_{a} f_{s} a_{a}^{3} \hfill \\ + \left( {k_{1}^{4} k_{2} - 5k_{1}^{2} k_{2}^{3} } \right)f_{s}^{2} a_{a}^{2} a_{s} + \left( {2k_{1}^{4} k_{2} - k_{1} k_{2}^{4} + 5k_{1}^{3} k_{2}^{2} + 2k_{1}^{2} k_{2}^{3} } \right)f_{a}^{2} a_{a}^{3} \hfill \\ - 4k_{1}^{3} k_{2}^{2} f_{a} f_{s} a_{s}^{2} a_{a} + \left( {k_{1}^{4} k_{2} - k_{1}^{2} k_{2}^{3} } \right)f_{s}^{2} a_{a}^{3} + \left( {2k_{1}^{3} k_{2}^{2} + 6k_{1}^{2} k_{2}^{3} } \right)f_{a} f_{s} a_{s}^{3} \hfill \\ + 4k_{1}^{2} k_{2}^{3} f_{a}^{2} a_{s}^{3} + 4k_{1}^{3} k_{2}^{2} f_{s}^{2} a_{s}^{2} a_{a} \hfill \\ \end{gathered} }{{a_{a} \theta_{a} \left[ {4k_{1} k_{2} a_{s} - \left( {k_{1} + k_{2} } \right)^{2} a_{a} } \right]^{2} }} \\ & \quad - NW_{ + s} + FR_{ + a} - IF_{ + a} \\ \end{aligned} $$

Under the equilibrium price, it is necessary to ensure that the driver supply of the platform is positive in both the self-operated mode and the aggregation mode (that is $0 < s_{ + i}^{ * } < 1,i = s,a$).

This constraint is equivalent to the constraint on indifference points (that is $0 < \omega_{ + 1} < \omega_{ + 2}^{{}} < 1$), According to the parameter setting given in this paper, it can be shown that $s_{ + s}^{ * } > 0,s_{ + a}^{ * } > 0$, Therefore, in order to ensure the non-negativity of the driver’s supply when the platform adopts the self-operated and aggregated business model, the following constraints should be satisfied:

1. $$\frac{{a_{a} }}{{a_{s} }} < \frac{{4k_{1} k_{2} }}{{\left( {k_{1} + k_{2}^{{}} } \right)^{2} }}or\frac{{a_{a} }}{{a_{s} }} \le \frac{{k_{2}^{{}} }}{{k_{1} }}$$.
2. $$\left[ {2k_{1} k_{2} a_{s} - \left( {k_{1} k_{2} + k_{2}^{2} } \right)a_{a} } \right]f_{a} - \left( {k_{1}^{2} - k_{1} k_{2} } \right)a_{a} f_{s} + \left( {k_{1} + k_{2} } \right)^{2} a_{a}^{2} > 4k{}_{1}k_{2} a_{a} a_{s}$$.

For the sake of simplicity, this paper defines the ratio of the driver’s satisfaction-sensitive cost under the unit service when the driver serves the self-operated business model and the aggregated business model as the driver’s heterogeneity ratio, which is recorded as $\frac{{a_{s} }}{{a_{a} }}$, The optimal results are shown in Proposition 3.

#### Proposition 3

In the self-operation + aggregation mode: the optimal driver reward, optimal supply and optimal profit of the platform are:

$$ \begin{gathered} h_{ + a}^{ * } = \frac{{\left( {k_{1}^{2} - k_{1} k_{2} } \right)a_{a} \theta_{f} + 2k_{1} k_{2} f_{a} \theta_{a} }}{{4k_{1} k_{2} a_{s} - \left( {k_{1} + k_{2} } \right)^{2} a_{a} }},\;h_{ + s}^{ * } = \frac{{\left( {k_{1}^{2} + k_{1} k_{2} } \right)\left( {f_{a} \theta_{a} - a_{a} \theta_{f} } \right) + 2k_{1} k_{2} \theta_{f} a_{s} }}{{4k_{1} k_{2} a_{s} - \left( {k_{1} + k_{2} } \right)^{2} a_{a} }}, \hfill \\ s_{ + a}^{ * } = \frac{{k_{1} \left( {k_{1} + k_{2} } \right)a_{a} f_{s} - 2k_{1} k_{2} a_{s} f_{a} }}{{a_{a} \left[ {\left( {k_{1} + k_{2} } \right)^{2} a_{a} - 4k_{1} k_{2} a_{s} } \right]}},\;s_{ + s}^{ * } = \frac{{2k_{1} k_{2} \theta_{f} - \left( {k_{1} k_{2} - k_{2}^{2} } \right)f_{a} }}{{4k_{1} k_{2} a_{s} - \left( {k_{1} + k_{2} } \right)^{2} a_{a} }}, \hfill \\ \end{gathered} $$

$$ \begin{aligned} P_{s + a}^{ * } & = \left( {k_{1} + k_{2} } \right)h_{ + s}^{ * } s_{ + s}^{ * } + \left( {k_{3} + k_{4} } \right)h_{ + a}^{ * } s_{ + a}^{ * } - NW_{ + s} \\ & = \frac{\begin{gathered} \left( {7k_{1}^{3} k_{2}^{2} + 2k_{1}^{2} k_{2}^{3} - 2k_{1}^{4} k_{2} + k_{1}^{{}} k_{2}^{4} } \right)f_{a} f_{s} a_{a}^{2} a_{s} + \left( {18k_{1}^{2} k_{2}^{3} + 3k_{1} k_{2}^{4} + 3k_{1}^{3} k_{2}^{2} } \right)f_{a}^{2} a_{a}^{2} a_{s} \hfill \\ + \left( {2k_{1}^{3} k_{2}^{2} - 2k_{1} k_{2}^{4} } \right)f_{a}^{2} a_{s}^{2} a_{a} + \left( {18k_{1}^{4} k_{2} - 9k_{1}^{3} k_{2}^{2} - 2k_{1}^{2} k_{2}^{3} - k_{1} k_{2}^{4} } \right)f_{a} f_{s} a_{a}^{3} \hfill \\ + \left( {k_{1}^{4} k_{2} - 5k_{1}^{2} k_{2}^{3} } \right)f_{s}^{2} a_{a}^{2} a_{s} + \left( {2k_{1}^{4} k_{2} - k_{1} k_{2}^{4} + 5k_{1}^{3} k_{2}^{2} + 2k_{1}^{2} k_{2}^{3} } \right)f_{a}^{2} a_{a}^{3} \hfill \\ - 4k_{1}^{3} k_{2}^{2} f_{a} f_{s} a_{s}^{2} a_{a} + \left( {k_{1}^{4} k_{2} - k_{1}^{2} k_{2}^{3} } \right)f_{s}^{2} a_{a}^{3} + \left( {2k_{1}^{3} k_{2}^{2} + 6k_{1}^{2} k_{2}^{3} } \right)f_{a} f_{s} a_{s}^{3} \hfill \\ + 4k_{1}^{2} k_{2}^{3} f_{a}^{2} a_{s}^{3} + 4k_{1}^{3} k_{2}^{2} f_{s}^{2} a_{s}^{2} a_{a} \hfill \\ \end{gathered} }{{a_{a} \theta_{a} \left[ {4k_{1} k_{2} a_{s} - \left( {k_{1} + k_{2} } \right)^{2} a_{a} } \right]^{2} }} \\ & \quad - NW_{ + s} + FR_{ + a} - IF_{ + a} \\ \end{aligned} $$

The heterogeneity ratio of the driver (i.e. $\frac{{a_{s} }}{{a_{a} }}$) to meet the following conditions:

1. $$\frac{{a_{a} }}{{a_{s} }} < \frac{{4k_{1} k_{2} }}{{\left( {k_{1} + k_{2}^{{}} } \right)^{2} }}or\frac{{a_{a} }}{{a_{s} }} \le \frac{{k_{2}^{{}} }}{{k_{1} }}$$.
2. $\left[ {2k_{1} k_{2} a_{s} - \left( {k_{1} k_{2} + k_{2}^{2} } \right)a_{a} } \right]f_{a} - \left( {k_{1}^{2} - k_{1} k_{2} } \right)a_{a} f_{s} + \left( {k_{1} + k_{2} } \right)^{2} a_{a}^{2} > 4k{}_{1}k_{2} a_{a} a_{s}$. (The proof is in the supplementary information).

**Figure 6:**
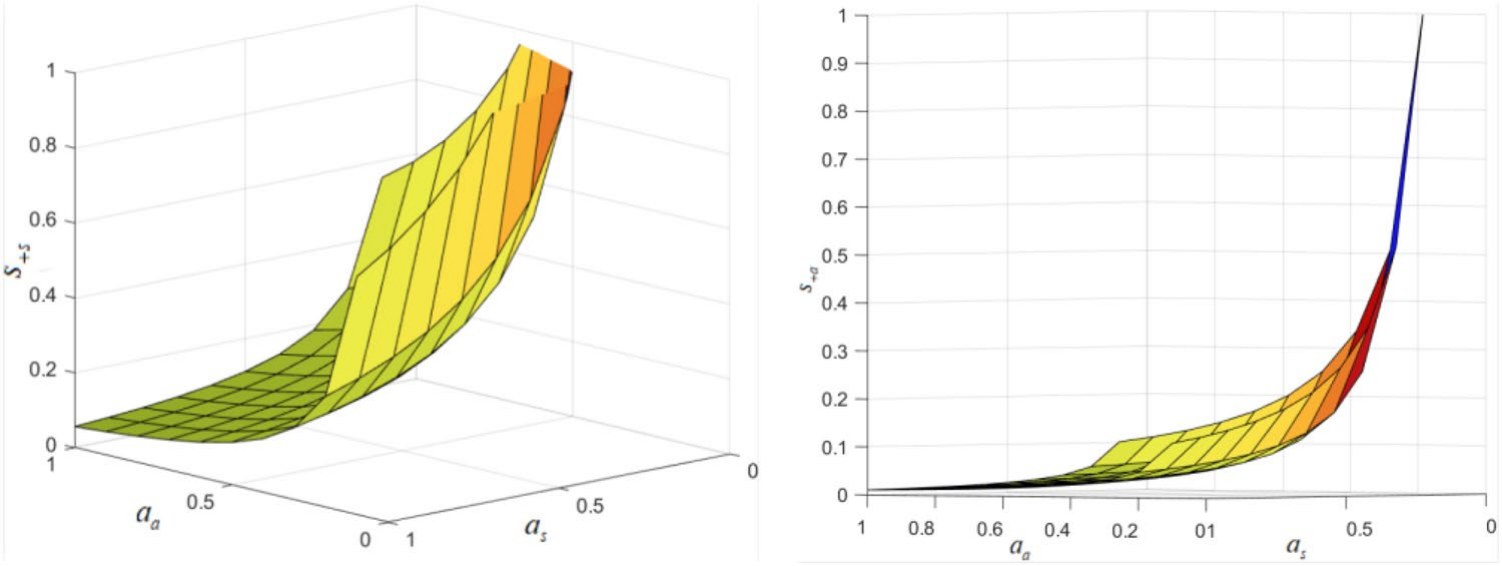
The impact of self-employed/aggregated drivers’ dissatisfaction on supply under self-employed + aggregated mode.

**Figure 7:**
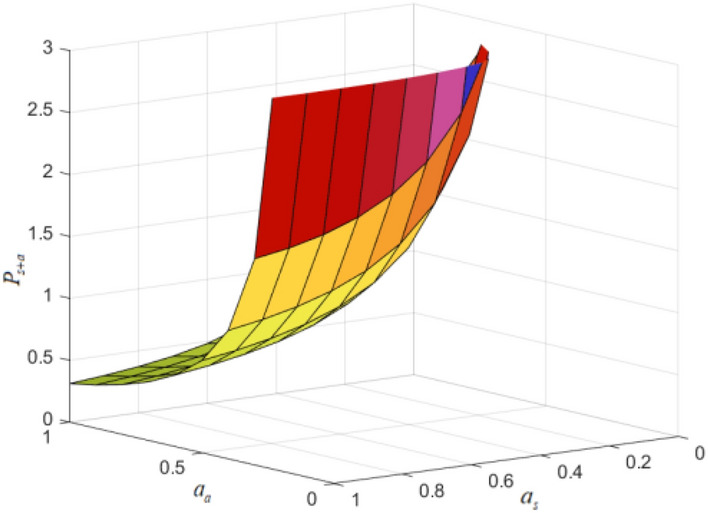
The impact of self-employed/aggregated drivers’ dissatisfaction on platform profits under self-employed + aggregated mode.

According to the optimization results, the driver’s disatisfaction has a more significant impact on the platform of the self-operated model. When the intrinsic value gap between the two business models of self-operation and aggregation is small, the platform can obtain higher profits. A typical example is Baidu Maps. It is now doing self-operated platforms and aggregation platforms at the same time, expanding the traffic of its own platform and improving the platform’s capacity. (As shown in Figs. 6 and 7, the examples are the same as above, $FR_{ + a} = 0.03,IF_{ + a} = 0.07,\mu = 0.02$).

## Optimal business model selection

In this paper, the optimal conditions of the three business models are listed. Table 2 lists the optimal reward of the driver, the optimal supply of the platform and the optimal profit of the platform under each business model. On this basis, this paper will compare the results of three different business models and obtain the optimal specific conditions of each business model.

**Table 2:** Optimal results under three business models.

Business model		Driver income	Platform supply	Platform’s profit
PSP		$h_{s}^{ * } = \frac{{f_{s} }}{2}$	$s_{s}^{ * } = \frac{{f_{s} }}{{2a_{s} }}$	$P_{psp}^{ * } = \left[ {\frac{{\beta \left( {1 - \gamma } \right)}}{\gamma } + \frac{{\left( {1 - \alpha } \right)\left( {1 - \beta } \right)}}{\alpha }} \right]\frac{{f_{s}^{2} }}{{4a_{s} }} - NW$
PAP		${\text{h}}_{{\text{a}}}^{ * } = \frac{{f_{a} }}{2}$	${\text{s}}_{a}^{ * } = \frac{{f_{a} }}{{2a_{a} }}$	$P_{PAP}^{ * } = \frac{{\mu \left( {1 - \alpha } \right)f_{a}^{2} }}{{4\alpha a_{a} }}$
S + A-P	Self-operating mod	$$h_{ + s}^{ * } = \frac{\begin{gathered} \left( {k_{1}^{2} + k_{1} k_{2} } \right)\left( {f_{a} \theta_{a} - a_{a} \theta_{f} } \right) \hfill \\ + 2k_{1} k_{2} \theta_{f} a_{s} \hfill \\ \end{gathered} }{{4k_{1} k_{2} a_{s} - \left( {k_{1} + k_{2} } \right)^{2} a_{a} }}$$	$s_{ + s}^{ * } = \frac{{2k_{1} k_{2} \theta_{f} - \left( {k_{1} k_{2} - k_{2}^{2} } \right)f_{a} }}{{4k_{1} k_{2} a_{s} - \left( {k_{1} + k_{2} } \right)^{2} a_{a} }}$	$$\begin{gathered} P_{s + a}^{ * } = \left( {k_{1} + k_{2} } \right)h_{ + s}^{ * } s_{ + s}^{ * } + \left( {k_{3} + k_{4} } \right)h_{ + a}^{ * } s_{ + a}^{ * } - NW_{ + s} \\ = \frac{\begin{gathered} \left( {7k_{1}^{3} k_{2}^{2} + 2k_{1}^{2} k_{2}^{3} - 2k_{1}^{4} k_{2} + k_{1}^{{}} k_{2}^{4} } \right)f_{a} f_{s} a_{a}^{2} a_{s} + \left( {18k_{1}^{2} k_{2}^{3} + 3k_{1} k_{2}^{4} + 3k_{1}^{3} k_{2}^{2} } \right)f_{a}^{2} a_{a}^{2} a_{s} \hfill \\ + \left( {2k_{1}^{3} k_{2}^{2} - 2k_{1} k_{2}^{4} } \right)f_{a}^{2} a_{s}^{2} a_{a} + \left( {18k_{1}^{4} k_{2} - 9k_{1}^{3} k_{2}^{2} - 2k_{1}^{2} k_{2}^{3} - k_{1} k_{2}^{4} } \right)f_{a} f_{s} a_{a}^{3} \hfill \\ + \left( {k_{1}^{4} k_{2} - 5k_{1}^{2} k_{2}^{3} } \right)f_{s}^{2} a_{a}^{2} a_{s} + \left( {2k_{1}^{4} k_{2} - k_{1} k_{2}^{4} + 5k_{1}^{3} k_{2}^{2} + 2k_{1}^{2} k_{2}^{3} } \right)f_{a}^{2} a_{a}^{3} \hfill \\ - 4k_{1}^{3} k_{2}^{2} f_{a} f_{s} a_{s}^{2} a_{a} + \left( {k_{1}^{4} k_{2} - k_{1}^{2} k_{2}^{3} } \right)f_{s}^{2} a_{a}^{3} + \left( {2k_{1}^{3} k_{2}^{2} + 6k_{1}^{2} k_{2}^{3} } \right)f_{a} f_{s} a_{s}^{3} \hfill \\ + 4k_{1}^{2} k_{2}^{3} f_{a}^{2} a_{s}^{3} + 4k_{1}^{3} k_{2}^{2} f_{s}^{2} a_{s}^{2} a_{a} \hfill \\ \end{gathered} }{{a_{a} \theta_{a} \left[ {4k_{1} k_{2} a_{s} - \left( {k_{1} + k_{2} } \right)^{2} a_{a} } \right]^{2} }} \\ - NW_{ + s} + FR_{ + a} - IF_{ + a} \\ \end{gathered}$$
	Aggregation mod	$$h_{ + a}^{ * } = \frac{\begin{gathered} \left( {k_{1}^{2} - k_{1} k_{2} } \right)a_{a} \theta_{f} \hfill \\ + 2k_{1} k_{2} f_{a} \theta_{a} \hfill \\ \end{gathered} }{{4k_{1} k_{2} a_{s} - \left( {k_{1} + k_{2} } \right)^{2} a_{a} }}$$	$s_{ + a}^{ * } = \frac{{k_{1} \left( {k_{1} + k_{2} } \right)a_{a} f_{s} - 2k_{1} k_{2} a_{s} f_{a} }}{{a_{a} \left[ {\left( {k_{1} + k_{2} } \right)^{2} a_{a} - 4k_{1} k_{2} a_{s} } \right]}}$	

By comparing the results, the platform obtains the optimal results under certain conditions. Including the driver’s service preference heterogeneity ratio $\left( {\frac{{a_{s} }}{{a_{a} }}} \right)$, Operating cost of self-operated platform W, The joining fee IF received by the aggregation platform, The dishonesty rate FR of the aggregation platform, The proposition below gives the optimal choice of the platform for the business model.

Proposition 4: (the choice of the optimal business model of the platform).

When all individual rationality constraints are satisfied, the following optimal result choices can be obtained:

- When $W_{ + s} + IF_{ + a}^{{}} < \overline{{W_{ + s} + IF_{ + a}^{{}} }}$ and $IF_{ + a} < \overline{{IF_{ + a} }}$, Pure self-operation mode is the best choice for the platform..
- When $FR{}_{ + a}^{{}} < \overline{{FR{}_{ + a}^{{}} }}$ and $\frac{{a_{s} }}{{a_{a} }} \in \varepsilon$, The self-management + aggregation business model is the optimal choice for the platform.
- When $FR_{ + a} < \overline{{FR_{ + a} }}^{\prime }$ and $W_{ + s} < \overline{{W_{ + s} }}$, Self-operated + aggregated business model is the best choice for the platform. (The proof is in the supplementary information).

Proposition 4 expounds the optimal business model selection of the platform based on the heterogeneity ratio of driver’s service preference, the operating cost of the self-operated platform, the franchise fee received by the aggregation platform and the dishonesty rate of the aggregation platform.

Proposition 4 shows the condition of pure self-management mode as the optimal choice of platform. $W_{ + s} + IF_{ + a}^{{}} < \overline{{W_{ + s} + IF_{ + a}^{{}} }}$ shows that it is unrealistic to reduce the operating cost by getting part of the aggregation platform from the small platform through the self-operation + aggregation business model, which will only lead to the maximization of the net profit of the platform, so the aggregation business model should be abandoned. $IF_{ + a} < \overline{{IF_{ + a} }}$ shows that the platform joining cost in the aggregation mode is too small to choose the aggregation mode. To sum up, in the case of a, the platform should choose a pure self-operation mode.

b shows the conditions of pure aggregation business model as the optimal choice of the platform. $FR{}_{ + a}^{{}} < \overline{{FR{}_{ + a}^{{}} }}$ shows that the dissatisfaction rate of the original consumers to the platform is very low in the aggregation mode. $\frac{{a_{s} }}{{a_{a} }} \in \varepsilon$ shows that the aggregation mode should be adopted when the driver is more sensitive to the heterogeneity of service quality, because the platform has low qualification requirements for the driver under this mode, and the barrier for the driver to carry out the industry is low. The driver will be more likely to accept the service quality and have a positive effect on improving the supply. At this time, the platform should adopt a pure aggregation business model.

c shows the conditions of self-operation + aggregation business model as the optimal choice of platform. $FR_{ + a} < \overline{{FR_{ + a} }}^{\prime }$ shows that in the aggregation mode, the original consumer’s dissatisfaction rate with the platform is very low. In this case, the aggregation mode is used to minimize the loss of sticky users, that is, customers can be maintained and revenue can be obtained. $W_{ + s} < \overline{{W_{ + s} }}$ shows that the operating cost of the platform is not high under the self-operated mode, and the self-operated mode can greatly improve the net profit. In summary, the platform should adopt a self-operated + aggregated business model when it can ensure user stickiness and maximize the loss of original users and maximize profits.

## Model extension

In this section, we analyze the changes of the two models by focusing on the different behaviors of drivers. We find that the results in the pure self-operated mode, the pure aggregation mode and the self-operated + aggregation mode are robust.

### Drivers who are strongly sensitive to service quality vs drivers who are weakly sensitive to service quality

Here, we extend the model to models with strong and weak sensitivity of drivers to service quality. Therefore, we assume that there are two special types of drivers, drivers with a ratio of $\eta$ are drivers who are highly sensitive to the quality of service and at the same time they will have a sensitive cost to the quality of service $a_{iq} (i = s,a)$, drivers with a ratio of $1 - \eta$ are drivers who are weakly sensitive to the quality of service and at the same time they will have a sensitive cost to the quality of service $a_{ir}$.

#### Comparison of service quality-sensitive drivers under PAP and PSP business models

Considering the case of a single business model, that is $i = s,a$, the driver’s utility model in this extended model is similar to 3.1 and 3.2, that is,

24 $$ \begin{gathered} u_{iq} = f_{i} - h_{i} - a{}_{iq}\omega \hfill \\ u_{ir} = f_{i} - h_{i} - a{}_{ir}\omega \hfill \\ \end{gathered} $$

We can conclude that the indifference points are $\omega_{iq} = \frac{{f_{i} - h_{i} }}{{a_{iq} }}$ and $\omega_{ir} = \frac{{f_{i} - h_{i} }}{{a_{ir} }}$, according to the indifference point, the driver’s supply can be obtained as $s_{i} = \eta \frac{{f_{i} - h_{i} }}{{a_{iq} }} + (1 - \eta )\frac{{f_{i} - h_{i} }}{{a_{ir} }}$. So the profit functions are $P_{psp} = (\beta \frac{1 - \gamma }{\gamma } + (1 - \beta )\frac{1 - \alpha }{\alpha })h_{s} (\eta \frac{{f_{s} - h{}_{s}}}{{a_{sq} }} + (1 - \eta )\frac{{f_{s} - h{}_{s}}}{{a_{sr} }}) - NW$ and $P_{pap} = \mu \frac{1 - \alpha }{\alpha }h_{a} (\eta \frac{{f_{a} - h{}_{a}}}{{a_{aq} }} + (1 - \eta )\frac{{f_{a} - h{}_{a}}}{{a_{ar} }}) - NFR + NIF$ respectively. Using the method in the basic model, we can get the optimal reward of the driver is $h_{i}^{ * } = \frac{{f_{i} }}{2}$, Bringing the optimal reward of the driver into the driver supply and platform profit function, the optimal supply and optimal profit of the platform driver can be obtained as follows:

25 $$ s_{s}^{ * } = \frac{{\eta f_{s} }}{{2a_{sq} }} + \frac{{(1 - \eta )f_{s} }}{{2a_{sr} }} $$

26 $$ P_{psp}^{ * } = (\beta \frac{1 - \gamma }{\gamma } + (1 - \beta )\frac{1 - \alpha }{\alpha })(\frac{{a_{sr} \eta + a_{sq} (1 - \eta )}}{{4a_{sq} a_{sr} }})f_{s}^{2} - NW $$

27 $$ s_{s}^{ * } = \frac{{\eta f_{s} }}{{2a_{sq} }} + \frac{{(1 - \eta )f_{s} }}{{2a_{sr} }} $$

28 $$ P_{pap}^{ * } = \mu \frac{1 - \alpha }{\alpha }(\frac{{a_{ar} \eta + a_{aq} (1 - \eta )}}{{4a_{aq} a_{ar} }})f_{a}^{2} - NIF + NFR $$

The basic utility of the driver to meet $f_{i} < 2a_{is}$, These studies show that the model is robust by considering drivers with different sensitivity to service quality. (The proof process is similar to 3.1 and 3.2).

#### The driver’s sensitivity to service quality under $P_{S + A}^{{}}$ business model

The self-operation + aggregation business model is more complicated than the pure self-operation model and the pure aggregation model. The driver’s utility model is similar to the basic model, which can be written as $u_{ + ij} = f_{i} - h_{ + i} - a_{ij} \omega ,i = s,a,j = q,r)$. By solving the indifference point equations, we obtain two indifference points $\omega_{ + sj} = \frac{{f_{s} - h_{ + s} + h_{ + a} }}{{a_{sj} - a_{aj} }}$ and $\omega_{ + aj} = \frac{{f_{a} - h_{ + a} }}{{a_{aj} }} - \frac{{f_{s} - h_{ + s} - f_{a} + h_{ + a} }}{{a_{sj} - a_{aj} }}$ respectively. So the driver’s supply can be obtained as

29 $$ s_{ + sj} = \eta \frac{{\theta_{f} - h_{ + a} + h_{ + a} }}{{\theta_{aq} }} + (1 - \eta )\frac{{\theta_{f} - h_{ + a} + h_{ + a} }}{{\theta_{ar} }} $$

30 $$ s_{ + aj} = \eta (\frac{{f_{a} - h_{ + a} }}{{a_{aq} }} - \frac{{\theta_{f} - h_{ + s} + h_{ + a} }}{{\theta_{aq} }}) + (1 - \eta )(\frac{{f_{a} - h_{ + a} }}{{a_{ar} }} - \frac{{\theta_{f} - h_{ + s} + h_{ + a} }}{{\theta_{ar} }}) $$

Substituting formulas (29) and (30) into formula (13), we can get

$$ \begin{aligned} p_{s + a} & = k_{1} \frac{{\eta \theta_{ar} + (1 - \eta )\theta_{aq} }}{{\theta_{ar} \theta_{aq} }}(\theta_{f} h_{ + s} - h_{ + s}^{2} + h_{ + s} h_{ + a} ) + k_{2} \frac{1 - \eta }{{a_{ar} \theta_{ar} }}(\theta_{ar} (h_{ + a} f_{a} - h_{ + a}^{2} ) - a_{ar} (\theta_{f} h_{ + a} + h_{ + a}^{2} - h_{ + s} h_{ + a} ) \\ & \quad + k_{2} \frac{\eta }{{a_{aq} \theta_{aq} }}(\theta_{aq} (h_{ + a} f_{a} - h_{ + a}^{2} ) - a_{aq} (\theta_{f} h_{ + a} + h_{ + a}^{2} - h_{ + s} h_{ + a} ) - NW_{ + s} + FR_{ + a} - IF_{ + a} \\ \end{aligned} $$

Therefore, the optimal reward of the driver can be obtained as $h_{ + s}^{ * } = \frac{{M_{1} M_{3} + 2M_{2} M_{4} \theta_{f} }}{{2M_{2} M_{4} - M_{1}^{2} }}$ and $h_{ + a}^{ * } = \frac{{M_{2} M_{3} + M_{1} M_{2} \theta_{f} }}{{2M_{2} M_{4} - M_{1}^{2} }}$, The optimal supply of the platform is

$$ s_{ + s}^{ * } = \eta \frac{{M_{2} (M_{3} - M_{1} ) + \theta_{f} M_{2} (M_{1} - M_{4} )}}{{\theta_{q} (2M_{2} M_{4} - M_{1}^{2} )}} + (1 - \eta )\frac{{M_{2} (M_{3} - M_{1} ) + \theta_{f} M_{2} (M_{1} - M_{4} )}}{{\theta_{r} (2M_{2} M_{4} - M_{1}^{2} )}} $$

$$ \begin{gathered} s_{ + a}^{ * } = \frac{{(\eta a_{ar} + (1 - \eta )a_{aq} )((2M_{2} M_{4} - M_{1}^{2} )f_{a} - M_{1} M_{2} \theta_{f} + M_{2} M_{3} )}}{{a_{aq} a_{ar} (2M_{2} M_{4} - M_{1}^{2} )}} - \hfill \\ {\kern 1pt} {\kern 1pt} {\kern 1pt} {\kern 1pt} {\kern 1pt} {\kern 1pt} {\kern 1pt} {\kern 1pt} {\kern 1pt} {\kern 1pt} {\kern 1pt} {\kern 1pt} {\kern 1pt} {\kern 1pt} {\kern 1pt} {\kern 1pt} {\kern 1pt} {\kern 1pt} {\kern 1pt} {\kern 1pt} {\kern 1pt} {\kern 1pt} {\kern 1pt} {\kern 1pt} {\kern 1pt} {\kern 1pt} {\kern 1pt} {\kern 1pt} {\kern 1pt} {\kern 1pt} {\kern 1pt} {\kern 1pt} {\kern 1pt} {\kern 1pt} {\kern 1pt} {\kern 1pt} {\kern 1pt} {\kern 1pt} \frac{{(\eta \theta_{r} + (1 - \eta )\theta_{q} )((M_{2} - M_{1}^{{}} )M_{1}^{{}} \theta_{f} + M_{1} M_{2} - M_{2} M_{3} )}}{{\theta_{q} \theta_{r} (2M_{2} M_{4} - M_{1}^{2} )}} \hfill \\ \end{gathered} $$

Which, $$M_{1} = \frac{{k_{1} (\eta \theta_{r} + (1 - \eta )\theta_{q} ) + k_{2} (\eta \theta_{r} + (1 - \eta )\theta_{q} )}}{{\theta_{r} \theta_{q} }}$$, $$M_{2} = \frac{{k_{1} (\eta \theta_{r} + (1 - \eta )\theta_{q} )}}{{\theta_{r} \theta_{q} }}$$, $$M_{3} = \frac{{k_{2} (1 - \eta )}}{{a_{ar} \theta_{r} }}(a_{sr} f_{a} - a_{ar} f_{s} ) + \frac{{k_{2} \eta }}{{a_{aq} \theta_{q} }}(a_{sq} f_{a} - a_{aq} f_{s} )$$, $$M_{4} = \frac{{k_{2} (1 - \eta )a_{sr} }}{{a_{ar} \theta_{r} }} + \frac{{k_{2} \eta a_{sq} }}{{a_{aq} \theta_{q} }}$$, $$\theta_{q} = a_{sq} - a_{aq}$$, $$\theta_{r} = a_{sr} - a_{ar}$$.

Therefore, the optimal profit of the platform is

$$ \begin{aligned} p_{s + a}^{ * } & = k_{1} \frac{{(\eta \theta_{r} + (1 - \eta )\theta_{q} )(M_{2} (M_{3} - M_{1} )) + \theta_{f} M_{2} (M_{1} - M_{4} )(M_{1} M_{3} + 2M_{2} M_{4} \theta_{f} )}}{{\theta_{r} \theta_{q} (2M_{2} M_{4} - M_{1}^{2} )^{2} }} + \\ & \quad 2k_{2} \frac{{M_{2} M_{4} (M_{1} \theta_{f} + M_{3} )(\eta a_{ar} + (1 - \eta )a_{aq} )((2M_{2} M_{4} - M_{1}^{2} )f_{a} - M_{1} M_{2} \theta_{f} + M_{2} M_{3} )}}{{2a_{aq} a_{ar} M_{4} (2M_{2} M_{4} - M_{1}^{2} )^{2} }} - - \\ & \quad 2k_{2} \frac{{M_{2} M_{4} (M_{1} \theta_{f} + M_{3} )(\eta \theta_{r} + (1 - \eta )\theta_{q} )(M_{1} \theta_{f} (M_{2} - M_{1}^{{}} ) - M_{3} M_{2} + M_{2} M_{1} )}}{{2\theta_{q} \theta_{r} M_{4} (2M_{2} M_{4} - M_{1}^{2} )^{2} }} \\ & \quad - NW_{ + s} + FR_{ + a} - IF_{ + a} \\ \end{aligned} $$

Similar to the basic model, this result still shows that when the platform decides to adopt the self-employed + aggregated business model, the platform should pay attention to reducing the quality of service to drivers in order to obtain greater profits. (The proof process is similar to 3.3).

### The situation when the driver is multi-homing

In the basic model, in order to make the model relatively simple, we assume that the driver does not have multi-homing behavior, but in real life, some drivers have multi-homing behavior, so we extend the results to the case where the driver has multi-homing behavior. We assume that $\delta$ driver does not have multi-homing behavior, and $1 - \delta$ drivers have multi-homing behavior and belong to n platforms at the same time. For the sake of simplicity of calculation, we use $M = \delta + n(1 - \delta )$ to discuss this situation based on self-operation + aggregation mode. Similar to the basic model, the supply function in this case is

31 $$ s_{ + s} = M\frac{{\theta_{f} - h_{ + s} + h_{ + a} }}{{\theta_{a} }} $$

32 $$ s_{ + a} = M(\frac{{f_{a} - h_{ + a} }}{{a_{a} }} + \frac{{\theta_{f} - h_{ + s} + h_{ + a} }}{{\theta_{a} }}) $$

Substituting Eq. (31) and Eq. (32) into Eq. (13), the profit function of the platform can be obtained as $p_{s + a} = \frac{{(k_{1} M\theta_{f} - k_{2} Ma_{a} )h_{ + s} - k_{1} Mh_{ + s}^{2} + \theta_{a}^{{}} k_{2} Mf_{a} + a_{a} k_{2} M\theta_{f} + k_{1} Mh_{ + s} h_{ + a} + (a_{a} - \theta_{a} )k_{2} Mh_{ + a} }}{{a_{a} \theta_{a} }}$.

Therefore, the optimal reward of the driver can be obtained as

$$ h_{ + a}^{ * } = \frac{{2k_{1} k_{2} M^{2} (\theta_{a} f_{a} + \theta_{f} a_{a} ) + (k_{1} M + k_{2} Ma_{a} )k_{1} M\theta_{f} }}{{4k_{1} k_{2} M^{2} a_{s} - (k_{1} M + k_{2} Ma_{a} )^{2} }} $$

$$ h_{ + s}^{ * } = \frac{{2k_{1}^{2} k_{2}^{2} \theta_{f} + (k_{1} M + k_{2} Ma_{a} )k_{2} M(\theta_{a} f_{a} + \theta_{f} a_{a} )}}{{4k_{1} k_{2} M^{2} a_{s} - (k_{1} M + k_{2} Ma_{a} )^{2} }} $$

So the optimal supply of the platform is

$$ \begin{aligned} s_{ + a}^{ * } & = \frac{{k_{1} k_{2} M^{2} (a_{s} + a_{a} )(f_{a} - \theta_{f} a_{a} ) - (k_{1} M + k_{2} Ma_{a} )(k_{1} Mf_{s} - k_{2} Ma_{a} f_{a} }}{{4k_{1} k_{2} M^{2} a_{s} a_{a} - a_{a} (k_{1} M + k_{2} Ma_{a} )^{2} }} \\ & \quad {\kern 1pt} + \frac{{k_{2} M^{2} (k_{1} - k_{2} a_{a} )(\theta_{a} f_{a} + \theta_{f} a_{a} ) + M^{2} (k_{1}^{2} M + 4k_{1} k_{2} - k_{2}^{2} a_{a}^{2} )\theta_{f} }}{{4k_{1} k_{2} M^{2} a_{s} \theta_{a} - \theta_{a} (k_{1} M + k_{2} Ma_{a} )^{2} }} \\ \end{aligned} $$

$$ s_{ + s}^{ * } = \frac{{2k_{1} k_{2} (2M^{2} a_{s} - k_{1} k_{2} ) + k_{2} M(k_{2} M - k_{1} M)(\theta_{a} f_{a} + \theta_{f} a_{a} ) - (k_{1}^{{}} M + k_{2}^{{}} Ma_{a}^{{}} )\theta_{f} }}{{4k_{1} k_{2} M^{2} a_{s} \theta_{a} - \theta_{a} (k_{1} M + k_{2} Ma_{a} )^{2} }} $$

So the optimal profit of the platform is

$$ \begin{gathered} p_{s + a}^{ * } = \frac{\begin{gathered} (2k_{1}^{2} k_{2} (2M^{2} a_{s} - k_{1} k_{2} ) - (k_{1} M + k_{2} Ma_{a} )k_{1} k_{2} a_{a} \theta_{f} + k_{1} k_{2} (k_{2} M - k_{1} M)(\theta_{a} f_{a} + \theta_{f} a_{a} )) \hfill \\ (2k_{1} k_{2} M^{2} \theta_{f} \theta_{a} + \theta_{a} (k_{1} M + k_{2} Ma_{a} )k_{2} M(\theta_{a} f_{a} + \theta_{f} a_{a} )) \hfill \\ \end{gathered} }{{\theta_{a} (4k_{1} k_{2} M^{2} a_{s} - (k_{1} M + k_{2} Ma{}_{a})^{2} }} \hfill \\ {\kern 1pt} {\kern 1pt} {\kern 1pt} {\kern 1pt} {\kern 1pt} {\kern 1pt} {\kern 1pt} {\kern 1pt} {\kern 1pt} {\kern 1pt} {\kern 1pt} {\kern 1pt} {\kern 1pt} {\kern 1pt} {\kern 1pt} {\kern 1pt} {\kern 1pt} {\kern 1pt} {\kern 1pt} {\kern 1pt} {\kern 1pt} {\kern 1pt} {\kern 1pt} {\kern 1pt} {\kern 1pt} {\kern 1pt} {\kern 1pt} + \frac{\begin{gathered} (2k_{1} k_{2}^{2} a_{a} \theta_{a} (\theta_{a} f_{a} + \theta_{f} a_{a} ) + k_{1} k_{2} Ma_{a} \theta_{a} \theta_{f} (k_{1} M + k_{2} Ma_{a} ))(k_{1} k_{2} \theta_{a} M^{2} ((a_{s} + a_{a} )f_{a} - \theta_{f} a_{a} ) - \hfill \\ \theta_{a} (k_{1} M + k_{2} Ma_{a} )(k_{1} Mf_{s} - k_{2} Ma_{a} f_{a} )) + (2k_{1} k_{2}^{2} a_{a} \theta_{a} (\theta_{a} f_{a} + \theta_{f} a_{a} ) + k_{1} k_{2} Ma_{a} \theta_{a} \theta_{f} \hfill \\ (k_{1} M + k_{2} Ma_{a} ))(k_{2}^{2} a_{a} \theta_{a} M^{2} (k_{1}^{{}} - k_{2} a_{a} )(\theta_{a} f_{a} + \theta_{f} a_{a} ) + k_{2} a_{a} \theta_{a} \theta_{f} M^{2} (k_{1}^{2} + 4k{}_{1}k_{2} - k_{2}^{2} a_{a}^{2} )) \hfill \\ \end{gathered} }{{a_{a} \theta_{a} (4k_{1} k_{2} M^{2} a_{s} - (k_{1} M + k_{2} Ma{}_{a})^{2} }} \hfill \\ \end{gathered} $$

By comparing this result with Proposition 3, we find that our results are robust. This result also shows that reducing the self-operation mode and aggregation mode is conducive to improving the service quality of drivers and improving the interests of the platform. (The proof process is similar to 3.3).

### The situation when there are heterogeneous passengers

In the above research, we only consider the supply side and do not consider the impact of the demand side on the platform profit. But in reality, the impact of passengers on the shared travel platform is also crucial, especially heterogeneous passengers. In the above, we mentioned that the pure aggregation platform has a loose audit of the driver, resulting in a relatively low qualification of the driver who joins the pure aggregation platform. For passengers, the service quality and price will be lower, so it is more sensitive to the price. Passengers who are not so sensitive to service quality generally choose the service of the pure aggregation platform. The pure self-operated platform will be more stringent for the driver’s audit, resulting in a relatively high qualification of the driver, and the quality of service and price will be higher for passengers, so it is more sensitive to the quality of service, and passengers who are less sensitive to the price will generally choose the service of the pure aggregation platform.

#### Comparison of PAP business model and PSP business model platform in the presence of heterogeneous passengers

When there are heterogeneous passengers who are more sensitive to price than service quality, it is more advantageous for the pure aggregation platform. The ultimate goal of the platform is still to maximize profits, that is, to maximize utility. We assume that the sensitivity coefficient of such passengers is $\xi_{a}$, Therefore, the utility function of the platform can be written as $u_{a} = (1 + \xi_{a} )p_{pap} - \xi_{a} p_{psp}$. When there are heterogeneous passengers who are more sensitive to service quality than price, it is more beneficial for the pure self-operated platform. The ultimate goal of the platform is still to maximize profits, that is, to maximize utility. We assume that the sensitivity coefficient of such passengers is $\xi_{s}$, Therefore, the utility function of the platform can be written as $u_{s} = (1 + \xi_{s} )p_{psp} - \xi_{s} p_{pap}$.

According to formula (3) and formula (6), we can get:

$$ u_{s} = (1 + \xi_{s} )(\beta \frac{1 - \gamma }{\gamma } + (1 - \beta )\frac{1 - \alpha }{\alpha }h_{s} \frac{{f_{s} - h_{s} }}{{a_{s} }} - NW) - \xi_{s} (\mu \frac{1 - \alpha }{\alpha }h_{a} \frac{{f_{a} - h_{a} }}{{a_{a} }} + NIF - NFR) $$

$$ u_{a} = (1 + \xi_{a} )(\mu \frac{1 - \alpha }{\alpha }h_{a} \frac{{f_{a} - h_{a} }}{{a_{a} }} + NIF - NFR) - \xi_{a} (\beta \frac{1 - \gamma }{\gamma } + (1 - \beta )\frac{1 - \alpha }{\alpha }h_{s} \frac{{f_{s} - h_{s} }}{{a_{s} }} - NW) $$

By solving $\frac{{\partial^{2} u_{s} }}{{\partial h_{s}^{2} }} < 0$ and $\frac{{\partial^{2} u_{a} }}{{\partial h_{a}^{2} }} < 0$, We can get the optimal solutions of $h_{a}$ and $h_{s}$ at the first order derivatives of $u_{a}$ and 4 $u_{s}$ to $h_{a}$ and $h_{s}$. So we can get:

$$ h_{s}^{*} = \frac{{f_{s} }}{2},\;s_{s}^{*} = \frac{{f_{s} }}{{2a_{s} }},\;P_{psp}^{ * } = \left[ {\frac{{\beta \left( {1 - \gamma } \right)}}{\gamma } + \frac{{\left( {1 - \alpha } \right)\left( {1 - \beta } \right)}}{\alpha }} \right]\frac{{f_{s}^{2} }}{{4a_{s} }} - NW $$

$$ h_{a}^{*} = \frac{{f_{a} }}{2},\;s_{a}^{*} = \frac{{f_{a} }}{{2a_{a} }},\;P_{PAP}^{ * } = \frac{{\mu \left( {1 - \alpha } \right)f_{a}^{2} }}{{4\alpha a_{a} }} + NIF - NFR $$

The basic utility of the driver to meet $f_{i} < 2a_{i}$.

These studies show that the model is robust by considering the existence of heterogeneous passengers. We find that when there are two types of heterogeneous passengers, the pure self-operation mode platform and the pure aggregation mode platform are compared. Passengers who are more sensitive to service quality than price are more inclined to pure self-operation mode platform, and passengers who are more sensitive to price than service quality are more inclined to pure aggregation mode platform.

#### Comparison of PSP business model and $P_{s + a}^{{}}$ business model platform when there are heterogeneous passengers

In this section, we study the comparison between the pure self-operation model and the self-operation + aggregation business model platform when there are heterogeneous passengers. The self-operation + aggregated business model platform includes services for two heterogeneous passengers. However, since the pure self-operated business model platform is dedicated to providing services for a heterogeneous passenger, in this section we assume that passengers who are more sensitive to the quality of service still prefer the pure self-operation business model platform. Therefore, the utility of the pure self-operation model platform is $u_{s} = (1 + \xi_{s} )p_{psp} - \xi_{s} p_{s + a}$. We assume that the sensitivity coefficient of passengers who are more sensitive to price is $\xi_{s + a}$, therefore, the utility of the self-operated + aggregated business model platform is $u_{s + a} = (1 + \xi_{s + a} )p_{s + a} - \xi_{s + a} p_{psp}$.

From Eq. (3) and Eq. (13), we can get:

$$ u_{s} = (1 + \xi_{s} )(A_{1} h_{s} \frac{{f_{s} - h_{s} }}{{a_{s} }} - NW) - \xi_{s} (A_{1} h_{s} \frac{{\theta_{f} - h_{s} + h_{a} }}{{\theta_{a}^{{}} }} + A_{2} h_{a} \frac{{f_{a} a_{s} - a_{a} f_{s} + a{}_{a}h_{s} - a_{s} h_{a} }}{{a_{a} \theta_{a} }} - NW + FR - IF) $$

$$ u_{s + a} = (1 + \xi_{s + a} )(A_{1} h_{s} \frac{{\theta_{f} - h_{s} + h_{a} }}{{\theta_{a}^{{}} }} + A_{2} h_{a} \frac{{f_{a} a_{s} - a_{a} f_{s} + a{}_{a}h_{s} - a_{s} h_{a} }}{{a_{a} \theta_{a} }} - NW + FR - IF) - \xi_{s + a} (A_{1} h_{s} \frac{{f_{s} - h_{s} }}{{a_{s} }} - NW) $$

which, $$A_{1} = \beta \frac{1 - \gamma }{\gamma } + \frac{(1 - \alpha )(1 - \beta )}{\alpha }$$, $$A_{2} = \frac{\mu (1 - \alpha )}{\alpha }$$.

(1) When the sensitivity coefficient of heterogeneous passengers is $\xi_{s}$.

At this time, we obtain $\frac{{\partial^{2} u_{s} }}{{\partial h_{s}^{2} }} < 0$_and_ $\frac{{\partial^{2} u_{s} }}{{\partial h_{a}^{2} }} < 0$ by solving $\frac{{\partial^{2} u_{s} }}{{\partial h_{s}^{2} }}$ and $\frac{{\partial^{2} u_{s} }}{{\partial h_{a}^{2} }}$ respectively. Therefore, the optimal solutions of $h_{s}$ and $h_{a}$ are obtained at the first derivative of $u_{s}$ to $h_{s}$ and $h_{a}$ respectively. Let $\frac{{\partial u_{s} }}{{\partial h_{s}^{{}} }} = 0$ and $\frac{{\partial u_{s} }}{{\partial h_{a}^{{}} }} = 0$ We can get the optimal reward of the driver is:

$$ h_{s}^{ * } = \frac{{2A_{2} a_{s} f_{s} (a_{s} - (1 + \xi_{s} )a_{a} ) + 2A_{2}^{{}} a_{a}^{2} \xi_{s} f_{a} - A_{2} \xi_{s} a_{s} (f_{a} a_{s} - a_{a} f_{s} )(1 + A_{2} )}}{{2A_{2} a_{s} (a_{s} - (1 + \xi )a_{a} ) - a_{a} a_{s} \xi_{s} (A_{2} - A_{1} \xi_{s} )(1 + A_{2} )}} $$

$$ h_{a}^{ * } = \frac{\begin{gathered} (2A_{2} a_{s} (a_{s} - (1 + \xi_{s} )a{}_{a}) - a_{a} a_{s} \xi_{s} (A_{2} - A{}_{1}\xi_{s} )(1 + A_{2} ))(f_{a} a_{s} - a_{a} f_{s} ) + \hfill \\ a_{a} (A_{2} - A_{1} \xi_{s} )(2a_{s} f_{s} (a_{s} - (1 + \xi_{s} )a_{a} ) + 2a_{a}^{2} \xi_{s} f_{a} - \xi_{s} a_{s} (f_{a} a_{s} - a_{a} f_{s} )(1 + A_{2} ) \hfill \\ \end{gathered} }{{2A_{2} a_{s}^{2} (a_{s} - (1 + \xi_{s} )a_{a} ) - a_{a} a_{s}^{2} \xi_{s} (A{}_{2} - A_{1} \xi_{s} )(1 + A_{2} )}} $$

The optimal supply of the pure self-operated platform can be obtained by bringing $h_{s}^{ * }$ into $s_{s} = \frac{{f_{s} - h_{s} }}{{a_{s} }}$:

$$ s_{s}^{*} = \frac{{2A_{2} a_{a}^{2} \xi_{s} f_{a} - a_{a}^{{}} \xi_{s} (A_{2} - A_{1} \xi_{s} )(1 + A_{2} )(f_{s} a_{s} - A_{2} )}}{{2A_{2}^{{}} a_{s}^{2} (a_{s} - (1 + \xi_{s} )a_{a} ) - a_{a} a{}_{s}^{2} \xi_{s} (A_{2} - A_{1} \xi_{s} )(1 + A_{2} )}} $$

Bringing $h_{s}^{ * }$ and $h_{a}^{ * }$ into formulas (11) and (12) can obtain the optimal supply of the aggregation platform

$$ s_{ + s}^{ * } = \frac{\begin{gathered} (2A_{2} a_{s} (a_{s} - (1 + \xi_{s} )a_{a} ) - (1 + A_{2} )\xi_{s} (a_{s} + A{}_{2}a_{a}^{2} + a_{a} a_{s} (A_{2} - A_{1} \xi_{s} )(f_{a} a{}_{s} - f_{s} a_{a} ) + \hfill \\ a_{a} a_{s} (A_{2} - A_{1} \xi {}_{s}^{{}} )(2f_{s} (a_{s} - (1 + \xi_{s} )a_{a} - \theta_{f}^{{}} a_{s} \xi_{s} (1 + A_{2} ) - 2A_{2} a_{s}^{2} (a_{s} - (1 + \xi_{s} )a_{a} )f_{a} + 2a_{a}^{2} \xi_{s} f_{a} (1 - A_{2} a_{s} ) \hfill \\ \end{gathered} }{{2\theta_{a} A_{2} a_{s}^{2} (a_{s} - (1 + \xi_{s} )a_{a} ) - a_{a} a_{s}^{2} \theta_{a} \xi_{s} (A_{2} - A_{1} \xi_{s} )(1 + A_{2} )}} $$

$$ s_{ + a}^{ * } = \frac{\begin{gathered} 2a_{a} a_{s} f_{s} (a_{s} - (1 + \xi {}_{s})a_{a} )(a_{a} (A_{2} - A_{1} \xi_{s} ) - A_{2} ) + \xi_{s} a_{s} (f_{a} a_{s} - a_{a} f_{s} )(1 + A{}_{2})(A_{2} a_{a} - 1) + \hfill \\ a_{s} (f_{a} a_{s} - a_{a} f_{s} )(2A_{2} (a_{s} - (1 + \xi_{s} )a_{a} ) - 2a_{a} \xi_{s} (A_{2} - A_{1} \xi_{s} (1 + A_{2} )) + 2A_{2} (a_{s} (a{}_{s} - (1 + \xi_{s} )a_{a} - a_{a}^{3} \xi_{s} f_{a} ) \hfill \\ \end{gathered} }{{2A_{2} a_{a} a_{s} \theta_{a} (a_{s} - (1 + \xi_{s} )a_{a} ) - a_{a}^{2} a_{s} \theta_{a} \xi_{s} (A_{2} - A_{1} \xi_{s} )(1 + A_{2} )}} $$

So we can get the optimal profits of the platform are respectively:

$$ \begin{gathered} p_{psp}^{ * } = A_{1} \frac{\begin{gathered} (2A_{2} a_{s} f_{s} (a_{s} - (1 + \xi_{s} )a_{a} ) + 2A_{2} a_{a}^{2} \xi_{s} f_{a} - A_{2} \xi_{s} a_{s} (f_{a} a_{s} - a_{a} f_{s} )(1 + A_{2} )) \hfill \\ (2A_{2} a_{a}^{2} \xi_{s} f_{a} - a_{a} \xi_{s} (A_{2} - A_{1} \xi_{s} )(1 + A_{2} )(f_{s} a_{s} - A_{2} )) \hfill \\ \end{gathered} }{{a_{s} (2A_{2} a_{s} (a_{s} - (1 + \xi_{s} )a_{a} ) - a_{a}^{{}} a_{s} \xi_{s} (A_{2} - A_{1} \xi_{s} )(1 + A_{2} ))^{2} }} \hfill \\ {\kern 1pt} {\kern 1pt} {\kern 1pt} {\kern 1pt} {\kern 1pt} {\kern 1pt} {\kern 1pt} {\kern 1pt} {\kern 1pt} {\kern 1pt} {\kern 1pt} {\kern 1pt} {\kern 1pt} {\kern 1pt} {\kern 1pt} {\kern 1pt} {\kern 1pt} {\kern 1pt} {\kern 1pt} {\kern 1pt} {\kern 1pt} {\kern 1pt} {\kern 1pt} {\kern 1pt} {\kern 1pt} {\kern 1pt} {\kern 1pt} {\kern 1pt} {\kern 1pt} {\kern 1pt} - NW \hfill \\ \end{gathered} $$

$$ \begin{gathered} p_{s + a}^{ * } = A_{1} \frac{\begin{gathered} (2A_{2} a_{s} f_{s} (a_{s} - (1 + \xi_{s} )a_{a} ) + 2A_{2} a_{a}^{2} \xi_{s} f_{a} - A_{2} \xi_{s} a_{s} (f_{a} a_{s} - a_{a} f_{s} )(1 + A_{2} ))((2A_{2} a_{s} (a_{s} - (1 + \xi_{s} )a_{a} ) \hfill \\ - (1 + A_{2} )\xi_{s} (a_{s} + A_{2} a_{a}^{2} + a_{a} a_{s} (A_{2} - A_{1} \xi_{s} )(f_{a} a_{s} - f_{s} a_{a} ) + a_{a} a_{s} (A_{2} - A_{1} \xi_{s} )(2f_{s} (a_{s} - (1 + \xi_{s} )a_{a} - \hfill \\ \theta_{f} a_{s} \xi_{s} (1 + A_{2} )) - 2A_{2} a_{s}^{2} (a_{s} - (1 + \xi_{s} )a_{a} )f_{a} + 2a_{a}^{2} \xi_{s} f_{a} (1 - A_{2} a_{s} )) \hfill \\ \end{gathered} }{{a_{s} \theta_{a} (2A_{2} a_{s} (a_{s} - (1 + \xi_{s} )a_{a} ) - a_{a}^{{}} a_{s} \xi_{s} (A_{2} - A_{1} \xi_{s} )(1 + A_{2} ))^{2} }} \hfill \\ {\kern 1pt} {\kern 1pt} {\kern 1pt} {\kern 1pt} {\kern 1pt} {\kern 1pt} {\kern 1pt} {\kern 1pt} {\kern 1pt} {\kern 1pt} {\kern 1pt} {\kern 1pt} {\kern 1pt} {\kern 1pt} {\kern 1pt} {\kern 1pt} {\kern 1pt} {\kern 1pt} {\kern 1pt} {\kern 1pt} {\kern 1pt} {\kern 1pt} {\kern 1pt} {\kern 1pt} {\kern 1pt} + A_{2} \frac{\begin{gathered} ((2A_{2} a_{s} (a_{s} - (1 + \xi_{s} )a{}_{a}) - a_{a} a_{s} \xi_{s} (A_{2} - A_{1} \xi {}_{s})(1 + A_{2} ))(f_{a} a_{s}^{{}} - f_{s} a_{a} ) + (a_{a} (A_{2} - A_{1} \xi_{s} )(2a_{s}^{{}} f_{s} (a_{s} \hfill \\ - (1 + \xi_{s} )a_{a} ) + 2a_{a}^{2} \xi_{s} f_{a} - \xi_{s} a_{s} (f_{a} a_{s} - a_{a} f_{s} )(1 + A_{2} ))(2a_{a} a_{s} (a_{s} - (1 + \xi_{s} )a_{a} )(a_{a} (A_{2} - A_{1} \xi_{s} ) - A_{2} ) \hfill \\ + \xi_{s} a_{s} (f_{a} a_{s} - a_{a} f_{s} )(1 + A_{2} )(A_{2} a_{a} - 1) + a_{s} (f_{a} a_{s} - a_{a} f_{s} )(2A_{2} (a_{s} - (1 + \xi_{s} )a_{a} ) - 2a_{a} \xi_{s} (A_{2} - A_{1} \xi {}_{s} \hfill \\ (1 + A_{2} )) + 2A_{2} (a_{s} (a_{s} - (1 + \xi_{s} )a_{a} - a_{a}^{3} \xi_{s} f_{a} )) \hfill \\ \end{gathered} }{{\theta_{a} (2A_{2} a_{s}^{2} (a_{s} - (1 + \xi )a_{a} ) - a_{a} a_{s}^{2} \xi_{s} (A_{2} - A_{1} \xi {}_{s})(1 + A_{2} ))^{2} }} \hfill \\ {\kern 1pt} {\kern 1pt} {\kern 1pt} {\kern 1pt} {\kern 1pt} {\kern 1pt} {\kern 1pt} {\kern 1pt} {\kern 1pt} {\kern 1pt} {\kern 1pt} {\kern 1pt} {\kern 1pt} {\kern 1pt} {\kern 1pt} {\kern 1pt} {\kern 1pt} {\kern 1pt} {\kern 1pt} {\kern 1pt} {\kern 1pt} {\kern 1pt} {\kern 1pt} {\kern 1pt} {\kern 1pt} {\kern 1pt} {\kern 1pt} - NW + NFR - NIF \hfill \\ \end{gathered} $$

The basic utility of the driver is to be satisfied $f_{i} < 2a_{i}$.

(2) When the sensitivity coefficient of heterogeneous passengers is $\xi_{s + a}$.

At this time, we obtain $\frac{{\partial^{2} u_{s + a} }}{{\partial h_{s}^{2} }} < 0$_and_ $\frac{{\partial^{2} u_{s + a} }}{{\partial h_{a}^{2} }} < 0$ by solving $\frac{{\partial^{2} u_{s} }}{{\partial h_{s}^{2} }}$ and $\frac{{\partial^{2} u_{s} }}{{\partial h_{a}^{2} }}$ respectively. Therefore, the optimal solutions of $h_{s}$ and $h_{a}$ are obtained at the first derivative of $u_{s + a}$ to $h_{s}$ and $h_{a}$ respectively. Let $\frac{{\partial u_{s + a} }}{{\partial h_{s}^{{}} }} = 0$ and $\frac{{\partial u_{s + a} }}{{\partial h_{a}^{{}} }} = 0$ We can get the optimal reward of the driver is:

$$ h_{s}^{ * } = \frac{{(1 + \xi_{s + a} )A_{2} (f_{a} a_{s} - a_{a} f_{s} )}}{{(1 + 2\xi_{s + a} )(2a_{s} - a_{a} (A_{1} + A_{2} )) - 2a_{a} \xi_{s + a} }} $$

$$ h_{a}^{ * } = \frac{{(f_{a} a{}_{s} - a_{a} f_{s} )((1 + \xi_{s + a} )((1 + \xi_{s + a} )a_{s} - \xi_{s + a} a_{a} ) - \xi_{s + a} a_{a} }}{{a_{s} (1 + 2\xi_{s + a} )(2a_{s} - a{}_{a}(A_{1} + A_{2} )) - 2a_{s} a_{a} \xi_{s + a} }} $$

The optimal supply of the pure self-operated platform can be obtained by bringing $h_{s}^{ * }$ into $s_{s} = \frac{{f_{s} - h_{s} }}{{a_{s} }}$:

$$ s_{s}^{*} = \frac{{(1 + \xi_{s + a} )(f_{s} (2a_{s} - a{}_{a}(A_{1} + A_{2} )) - (f{}_{a}a_{s} - f_{s} a_{a} )) - 2a_{a} f_{s} \xi_{s + a} }}{{a_{s} (1 + 2\xi_{s + a} )(2a_{s} - a_{a} (A_{1} + A_{2} )) - 2a_{a} a_{s} \xi_{s + a} }} $$

Bringing $h_{s}^{ * }$ and $h_{a}^{ * }$ into formulas (11) and (12) can obtain the optimal supply of the Self-operated + aggregated business model platform:

$$ s_{ + s}^{ * } = \frac{\begin{gathered} (2A_{2} a_{s} (a_{s} - (1 + \xi_{s} )a_{a} ) - (1 + A_{2} )\xi_{s} (a_{s} + A{}_{2}a_{a}^{2} + a_{a} a_{s} (A_{2} - A_{1} \xi_{s} )(f_{a} a{}_{s} - f_{s} a_{a} ) + \hfill \\ a_{a} a_{s} (A_{2} - A_{1} \xi {}_{s}^{{}} )(2f_{s} (a_{s} - (1 + \xi_{s} )a_{a} - \theta_{f}^{{}} a_{s} \xi_{s} (1 + A_{2} ) - 2A_{2} a_{s}^{2} (a_{s} - (1 + \xi_{s} )a_{a} )f_{a} + 2a_{a}^{2} \xi_{s} f_{a} (1 - A_{2} a_{s} ) \hfill \\ \end{gathered} }{{2\theta_{a} A_{2} a_{s}^{2} (a_{s} - (1 + \xi_{s} )a_{a} ) - a_{a} a_{s}^{2} \theta_{a} \xi_{s} (A_{2} - A_{1} \xi_{s} )(1 + A_{2} )}} $$

$$ s_{ + a}^{ * } = \frac{\begin{gathered} 2a_{a} a_{s} f_{s} (a_{s} - (1 + \xi {}_{s})a_{a} )(a_{a} (A_{2} - A_{1} \xi_{s} ) - A_{2} ) + \xi_{s} a_{s} (f_{a} a_{s} - a_{a} f_{s} )(1 + A{}_{2})(A_{2} a_{a} - 1) + \hfill \\ a_{s} (f_{a} a_{s} - a_{a} f_{s} )(2A_{2} (a_{s} - (1 + \xi_{s} )a_{a} ) - 2a_{a} \xi_{s} (A_{2} - A_{1} \xi_{s} (1 + A_{2} )) + 2A_{2} (a_{s} (a{}_{s} - (1 + \xi_{s} )a_{a} - a_{a}^{3} \xi_{s} f_{a} ) \hfill \\ \end{gathered} }{{2A_{2} a_{a} a_{s} \theta_{a} (a_{s} - (1 + \xi_{s} )a_{a} ) - a_{a}^{2} a_{s} \theta_{a} \xi_{s} (A_{2} - A_{1} \xi_{s} )(1 + A_{2} )}} $$

So we can get the optimal profits of the platform are respectively:

$$ p_{psp}^{*} = A_{1} \frac{{A_{2} (1 + \xi {}_{s + a})(f_{a} a_{s} - f{}_{s}a_{a} )((1 + \xi {}_{s + a})(f_{s} (2a_{s} - a_{a} (A_{1} + A_{2} )) - (f{}_{a}a_{s} - f_{s} a{}_{a})) - 2a_{a} f_{s} \xi {}_{s + a})}}{{a_{s} ((1 + 2\xi {}_{s + a})(2a_{s} - a_{a} (A_{1} + A_{2} )) - 2a_{a} \xi {}_{s + a})^{2} }} $$

$$ \begin{gathered} p_{s + a}^{*} = A_{1} \frac{\begin{gathered} A_{2} (1 + \xi {}_{s + a})(f_{a} a_{s} - f_{s} a_{a} )(\theta_{f} a_{s} (1 + 2\xi {}_{s + a})(2a_{s} - a_{a} (A_{1} + A_{2} )) \hfill \\ - 2a_{s} \theta_{f} a{}_{a}\xi {}_{s + a} + \xi {}_{s + a}((\xi {}_{s + a} + 1)a_{s} - (1 + 2\xi {}_{s + a})a_{a} )) \hfill \\ \end{gathered} }{{a_{s} ((1 + 2\xi {}_{s + a})(2a_{s} - a_{a} (A_{1}^{{}} + A_{2} )) - 2a_{a} \xi {}_{s + a})_{2} }} \hfill \\ {\kern 1pt} {\kern 1pt} {\kern 1pt} {\kern 1pt} {\kern 1pt} {\kern 1pt} {\kern 1pt} {\kern 1pt} {\kern 1pt} {\kern 1pt} {\kern 1pt} {\kern 1pt} {\kern 1pt} {\kern 1pt} {\kern 1pt} {\kern 1pt} {\kern 1pt} {\kern 1pt} {\kern 1pt} {\kern 1pt} {\kern 1pt} {\kern 1pt} {\kern 1pt} {\kern 1pt} {\kern 1pt} {\kern 1pt} {\kern 1pt} {\kern 1pt} {\kern 1pt} + A_{2} \frac{\begin{gathered} ((f_{a} a{}_{s} - a_{a} f_{s} )((1 + \xi_{s + a} )((1 + \xi_{s + a} )a_{s}^{{}} - \xi_{s + a} a_{a} ) - a_{a} \xi_{s + a} )) \hfill \\ ((f_{a} a{}_{s} - a_{a} f_{s} )((1 + 2\xi_{s + a} )(1 - \xi_{s + a} - A_{1} - A_{2} )a_{a} + (1 + \xi_{s + a} )a_{s} ) - 3a_{a} \xi_{s + a} ) \hfill \\ \end{gathered} }{{a_{s} a_{a} \theta_{a} ((1 + 2\xi_{s + a} )(2a_{s} - a{}_{a}(A_{1} + A_{2} )) - 2a_{a} \xi_{s + a} )^{2} }} \hfill \\ {\kern 1pt} {\kern 1pt} {\kern 1pt} {\kern 1pt} {\kern 1pt} {\kern 1pt} {\kern 1pt} {\kern 1pt} {\kern 1pt} {\kern 1pt} {\kern 1pt} {\kern 1pt} {\kern 1pt} {\kern 1pt} {\kern 1pt} {\kern 1pt} {\kern 1pt} {\kern 1pt} {\kern 1pt} {\kern 1pt} {\kern 1pt} {\kern 1pt} {\kern 1pt} {\kern 1pt} {\kern 1pt} {\kern 1pt} {\kern 1pt} {\kern 1pt} {\kern 1pt} {\kern 1pt} {\kern 1pt} {\kern 1pt} - NW + FR - IF \hfill \\ \end{gathered} $$

The basic utility of the driver is to be satisfied $f_{i} < 2a_{i}$.

By considering heterogeneous passengers, we can see that the model is robust. We find that when there are two types of heterogeneous passengers, compared with the pure self-operation mode platform, the self-operation + aggregation operation mode platform is more inclined to the pure self-operation mode platform than the passengers whose price is more sensitive to service quality, and the passengers whose service quality is more sensitive to price are more inclined to the self-operation + aggregation operation mode platform than the passengers whose service quality is more sensitive to price. Because although passengers who are more sensitive to the quality of service can meet their needs when they need services, the platform of the two business models can meet their needs, but the platform of the pure self-management model can reduce the time cost of selection.

#### Comparison of PAP business model and $P_{s + a}^{{}}$ business model platform when there are heterogeneous passengers

In this section, we study the comparison between the pure aggregation business model and the self-operated + aggregation business model platform when there are heterogeneous passengers. The self-operation + aggregation business model platform has services for two heterogeneous passengers at the same time. However, since the pure aggregation business model platform is committed to providing services for a heterogeneous passenger, in this section we assume that passengers who are more sensitive to prices still prefer the pure aggregation business model platform. Therefore, the utility of the pure aggregation business model platform is $u_{a} = (1 + \xi_{a} )p_{pap} - \xi_{a} p_{s + a}$. The utility of the self-operated + aggregated business model platform is $u_{s + a} = (1 + \xi_{s + a} )p_{s + a} - \xi_{s + a} p_{pap}$.

By forms (3) and (13) we can get:

$$ u_{a} = (1 + \xi_{a} )A_{2} h_{a} \frac{{f_{a} - h_{a} }}{{a_{a} }} - \xi_{a} (A_{1} h_{s} \frac{{\theta_{f} - h_{s} + h_{a} }}{{\theta_{a}^{{}} }} + A_{2} h_{a} \frac{{f_{a} a_{s} - a_{a} f_{s} + a{}_{a}h_{s} - a_{s} h_{a} }}{{a_{a} \theta_{a} }} - NW + FR - IF) $$

$$ u_{s + a} = (1 + \xi_{s + a} )(A_{1} h_{s} \frac{{\theta_{f} - h_{s} + h_{a} }}{{\theta_{a}^{{}} }} + A_{2} h_{a} \frac{{f_{a} a_{s} - a_{a} f_{s} + a{}_{a}h_{s} - a_{s} h_{a} }}{{a_{a} \theta_{a} }} - NW + FR - IF) - \xi_{s + a} A_{2} h_{a} \frac{{f_{a} - h_{a} }}{{a_{a} }} $$

which, $$A_{1} = \beta \frac{1 - \gamma }{\gamma } + \frac{(1 - \alpha )(1 - \beta )}{\alpha }$$, $$A_{2} = \frac{\mu (1 - \alpha )}{\alpha }$$.

(1) When the sensitivity coefficient of heterogeneous passengers is $\xi_{a}$.

At this time, we obtain $\frac{{\partial^{2} u_{a} }}{{\partial h_{s}^{2} }} < 0$_and_ $\frac{{\partial^{2} u_{a} }}{{\partial h_{a}^{2} }} < 0$ by solving $\frac{{\partial^{2} u_{s} }}{{\partial h_{s}^{2} }}$ and $\frac{{\partial^{2} u_{s} }}{{\partial h_{a}^{2} }}$ respectively. Therefore, the optimal solutions of $h_{s}$ and $h_{a}$ are obtained at the first derivative of $u_{a}$ to $h_{s}$ and $h_{a}$ respectively. Let $\frac{{\partial u_{a} }}{{\partial h_{s}^{{}} }} = 0$ and $\frac{{\partial u_{a} }}{{\partial h_{a}^{{}} }} = 0$ We can get the optimal reward of the driver is:

$$ h_{s}^{*} = \frac{{\theta_{f} A_{1} A_{2} (a_{s} (1 - 2\xi_{a} ) - a_{a} (1 - \xi_{a} )) + (A_{1} + A_{2} )(A_{2} f_{a} (a_{s} - (1 + \xi_{a} )a{}_{a}) + \xi_{a} a_{a} f_{s} }}{{2A_{1} A{}_{2}(a_{s} (1 - 2\xi_{a} ) - a_{a} (1 - \xi_{a} )) + (A_{1} + A_{2} )^{2} a_{a} \xi_{a} }} $$

$$ h_{a}^{*} = \frac{{2A_{1} A_{2} f_{a} (a_{s} (1 + \xi_{a} )a_{a} ) + (A_{1} \xi_{a} a_{a} (2f_{a} - \theta_{f}^{{}} (A_{1} + A_{2} ))}}{{2A_{1} A{}_{2}(a_{s} (1 - 2\xi_{a} ) - a_{a} (1 - \xi_{a} )) + (A_{1} + A_{2} )^{2} a_{a} \xi_{a} }} $$

The optimal supply of the pure aggregation business model can be obtained by bringing $h_{s}^{ * }$ into $s_{s} = \frac{{f_{s} - h_{s} }}{{a_{s} }}$:

$$ s_{a}^{*} = \frac{{4A_{1} A_{2} f_{a} \xi_{a} (a_{s} - a_{a} ) + a_{a} \xi_{a} (A_{1} + A_{2} )(f_{a} (2A_{1} + A_{2} ) - A_{1} f_{s} }}{{2A_{1} A{}_{2}a_{a} (a_{s} (1 - 2\xi_{a} ) - a_{a} (1 - \xi_{a} )) + (A_{1} + A_{2} )^{2} a_{a}^{2} \xi_{a} }} $$

Bringing $h_{s}^{ * }$ and $h_{a}^{ * }$ into formulas (11) and (12) can obtain the optimal supply of the self-operated + aggregated business model platform:

$$ s_{ + s}^{*} = \frac{\begin{gathered} \theta_{f} A_{1} A_{2} (a_{s} (1 - 2\xi_{a} ) - a_{a} (1 - \xi_{a} )) + \theta_{f} a_{a} \xi_{a} (A_{1} + A_{2} )^{2} + 2A_{1} A_{2} f_{a} (a_{s} - (1 + \xi_{a} )a_{a} ) \hfill \\ + A_{1} \xi_{a} a_{a} (2f_{a} - \theta_{f} )(A_{1} + A_{2} ) + (A_{1} + A_{2} )(A_{2} f_{a} (a_{s}^{{}} - (1 + \xi_{a} )a_{a} + \xi_{a} a_{a} f_{s} ) \hfill \\ \end{gathered} }{{2A_{1} A{}_{2}\theta_{a} (a_{s} (1 - 2\xi_{a} ) - a_{a} (1 - \xi_{a} )) + (A_{1} + A_{2} )^{2} \theta_{a} a_{a}^{{}} \xi_{a} }} $$

$$ s_{ + s}^{*} = \frac{\begin{gathered} A_{1} A_{2} (a_{s} (1 - 2\xi_{a} ) - a_{a} (1 - \xi_{a} ))(2(f_{a} a_{s} - f_{s} a_{a} ) + a_{a} \theta_{f} ) + A_{2} f_{a} (a_{s} - (1 + \xi_{a} )a_{a} )(a_{a} (A_{1} + A_{2} ) - 2a_{s} A_{1} + \hfill \\ (A_{1} + A_{2} )a_{a} \xi_{a} ((f_{a} a_{s} - f_{s} a{}_{a})(A_{1} + A_{2} ) + a_{a} f_{s} ) - a_{s} A_{1} a_{a} \xi_{a} (2f_{a} - \theta_{f} (A_{1} + A_{2} )) \hfill \\ \end{gathered} }{{2A_{1} A{}_{2}a_{a} \theta_{a} (a_{s} (1 - 2\xi_{a} ) - a_{a} (1 - \xi_{a} )) + (A_{1} + A_{2} )^{2} \theta_{a} a_{a}^{2} \xi_{a} }} $$

So we can get the optimal profits of the platform are respectively:

$$ p_{pap}^{*} = A_{2} \frac{\begin{gathered} (4A_{1} A_{2} f_{a} \xi_{a} (a_{s} - a_{a} ) + a_{a} \xi_{a} (A_{1} + A_{2} )(f_{a} (2A_{1} + A_{2} ) - A_{1} f_{s} ) \hfill \\ (2A_{1} A_{2} f_{a} (a_{s} - (1 + \xi_{a} )a_{a} ) + A_{1} \xi_{a} a_{a} (2f_{a} - \theta_{f} (A_{1} + A_{2} ))) \hfill \\ \end{gathered} }{{a_{a} (2A_{1} A{}_{2}(a_{s} (1 - 2\xi_{a} ) - a_{a} (1 - \xi_{a} )) + (A_{1} + A_{2} )^{2} a_{a}^{{}} \xi_{a} )^{2} }} + NIF - NFR $$

$$ \begin{gathered} p_{s + a}^{ * } = A_{1} \frac{\begin{gathered} (2A_{2} a_{s} f_{s} (a_{s} - (1 + \xi_{s} )a_{a} ) + 2A_{2} a_{a}^{2} \xi_{s} f_{a} - A_{2} \xi_{s} a_{s} (f_{a} a_{s} - a_{a} f_{s} )(1 + A_{2} ))((2A_{2} a_{s} (a_{s} - (1 + \xi_{s} )a_{a} ) \hfill \\ - (1 + A_{2} )\xi_{s} (a_{s} + A_{2} a_{a}^{2} + a_{a} a_{s} (A_{2} - A_{1} \xi_{s} )(f_{a} a_{s} - f_{s} a_{a} ) + a_{a} a_{s} (A_{2} - A_{1} \xi_{s} )(2f_{s} (a_{s} - (1 + \xi_{s} )a_{a} - \hfill \\ \theta_{f} a_{s} \xi_{s} (1 + A_{2} )) - 2A_{2} a_{s}^{2} (a_{s} - (1 + \xi_{s} )a_{a} )f_{a} + 2a_{a}^{2} \xi_{s} f_{a} (1 - A_{2} a_{s} )) \hfill \\ \end{gathered} }{{a_{s} \theta_{a} (2A_{2} a_{s} (a_{s} - (1 + \xi_{s} )a_{a} ) - a_{a}^{{}} a_{s} \xi_{s} (A_{2} - A_{1} \xi_{s} )(1 + A_{2} ))^{2} }} \hfill \\ {\kern 1pt} {\kern 1pt} {\kern 1pt} {\kern 1pt} {\kern 1pt} {\kern 1pt} {\kern 1pt} {\kern 1pt} {\kern 1pt} {\kern 1pt} {\kern 1pt} {\kern 1pt} {\kern 1pt} {\kern 1pt} {\kern 1pt} {\kern 1pt} {\kern 1pt} {\kern 1pt} {\kern 1pt} {\kern 1pt} {\kern 1pt} {\kern 1pt} {\kern 1pt} {\kern 1pt} {\kern 1pt} + A_{2} \frac{\begin{gathered} ((2A_{2} a_{s} (a_{s} - (1 + \xi_{s} )a{}_{a}) - a_{a} a_{s} \xi_{s} (A_{2} - A_{1} \xi {}_{s})(1 + A_{2} ))(f_{a} a_{s}^{{}} - f_{s} a_{a} ) + (a_{a} (A_{2} - A_{1} \xi_{s} )(2a_{s}^{{}} f_{s} (a_{s} \hfill \\ - (1 + \xi_{s} )a_{a} ) + 2a_{a}^{2} \xi_{s} f_{a} - \xi_{s} a_{s} (f_{a} a_{s} - a_{a} f_{s} )(1 + A_{2} ))(2a_{a} a_{s} (a_{s} - (1 + \xi_{s} )a_{a} )(a_{a} (A_{2} - A_{1} \xi_{s} ) - A_{2} ) \hfill \\ + \xi_{s} a_{s} (f_{a} a_{s} - a_{a} f_{s} )(1 + A_{2} )(A_{2} a_{a} - 1) + a_{s} (f_{a} a_{s} - a_{a} f_{s} )(2A_{2} (a_{s} - (1 + \xi_{s} )a_{a} ) - 2a_{a} \xi_{s} (A_{2} - A_{1} \xi {}_{s} \hfill \\ (1 + A_{2} )) + 2A_{2} (a_{s} (a_{s} - (1 + \xi_{s} )a_{a} - a_{a}^{3} \xi_{s} f_{a} )) \hfill \\ \end{gathered} }{{\theta_{a} (2A_{2} a_{s}^{2} (a_{s} - (1 + \xi )a_{a} ) - a_{a} a_{s}^{2} \xi_{s} (A_{2} - A_{1} \xi {}_{s})(1 + A_{2} ))^{2} }} \hfill \\ {\kern 1pt} {\kern 1pt} {\kern 1pt} {\kern 1pt} {\kern 1pt} {\kern 1pt} {\kern 1pt} {\kern 1pt} {\kern 1pt} {\kern 1pt} {\kern 1pt} {\kern 1pt} {\kern 1pt} {\kern 1pt} {\kern 1pt} {\kern 1pt} {\kern 1pt} {\kern 1pt} {\kern 1pt} {\kern 1pt} {\kern 1pt} {\kern 1pt} {\kern 1pt} {\kern 1pt} {\kern 1pt} {\kern 1pt} {\kern 1pt} - NW + NFR - NIF \hfill \\ \end{gathered} $$

The basic utility of the driver is to be satisfied $f_{i} < 2a_{i}$.

(2) When the sensitivity coefficient of heterogeneous passengers is $\xi_{s + a}$.

At this time, we obtain $\frac{{\partial^{2} u_{s + a} }}{{\partial h_{s}^{2} }} < 0$_and_ $\frac{{\partial^{2} u_{s + a} }}{{\partial h_{a}^{2} }} < 0$ by solving $\frac{{\partial^{2} u_{s + a} }}{{\partial h_{s}^{2} }}$ and $\frac{{\partial^{2} u_{s} }}{{\partial h_{a}^{2} }}$ respectively. Therefore, the optimal solutions of $h_{s}$ and $h_{a}$ are obtained at the first derivative of $u_{s + a}$ to $h_{s}$ and $h_{a}$ respectively. Let $\frac{{\partial u_{s + a} }}{{\partial h_{s}^{{}} }} = 0$ and $\frac{{\partial u_{s + a} }}{{\partial h_{a}^{{}} }} = 0$ We can get the optimal reward of the driver is:

$$ h_{s}^{ * } = \frac{{(1 + \xi_{s + a} )A_{2} (f_{a} a_{s} - a_{a} f_{s} )}}{{(1 + 2\xi_{s + a} )(2a_{s} - a_{a} (A_{1} + A_{2} )) - 2a_{a} \xi_{s + a} }} $$

$$ h_{a}^{ * } = \frac{{(f_{a} a{}_{s} - a_{a} f_{s} )((1 + \xi_{s + a} )((1 + \xi_{s + a} )a_{s} - \xi_{s + a} a_{a} ) - \xi_{s + a} a_{a} }}{{a_{s} (1 + 2\xi_{s + a} )(2a_{s} - a{}_{a}(A_{1} + A_{2} )) - 2a_{s} a_{a} \xi_{s + a} }} $$

The optimal supply of the pure aggregation business model can be obtained by bringing $h_{s}^{ * }$ into $s_{s} = \frac{{f_{s} - h_{s} }}{{a_{s} }}$:

$$ s_{s}^{*} = \frac{{(1 + \xi_{s + a} )(f_{s} (2a_{s} - a{}_{a}(A_{1} + A_{2} )) - (f{}_{a}a_{s} - f_{s} a_{a} )) - 2a_{a} f_{s} \xi_{s + a} }}{{a_{s} (1 + 2\xi_{s + a} )(2a_{s} - a_{a} (A_{1} + A_{2} )) - 2a_{a} a_{s} \xi_{s + a} }} $$

Bringing $h_{s}^{ * }$ and $h_{a}^{ * }$ into formulas (11) and (12) can obtain the optimal supply of the self-operated + aggregated business model platform:

$$ s_{ + s}^{ * } = \frac{\begin{gathered} (2A_{2} a_{s} (a_{s} - (1 + \xi_{s} )a_{a} ) - (1 + A_{2} )\xi_{s} (a_{s} + A{}_{2}a_{a}^{2} + a_{a} a_{s} (A_{2} - A_{1} \xi_{s} )(f_{a} a{}_{s} - f_{s} a_{a} ) + \hfill \\ a_{a} a_{s} (A_{2} - A_{1} \xi {}_{s}^{{}} )(2f_{s} (a_{s} - (1 + \xi_{s} )a_{a} - \theta_{f}^{{}} a_{s} \xi_{s} (1 + A_{2} ) - 2A_{2} a_{s}^{2} (a_{s} - (1 + \xi_{s} )a_{a} )f_{a} + 2a_{a}^{2} \xi_{s} f_{a} (1 - A_{2} a_{s} ) \hfill \\ \end{gathered} }{{2\theta_{a} A_{2} a_{s}^{2} (a_{s} - (1 + \xi_{s} )a_{a} ) - a_{a} a_{s}^{2} \theta_{a} \xi_{s} (A_{2} - A_{1} \xi_{s} )(1 + A_{2} )}} $$

$$ s_{ + a}^{ * } = \frac{\begin{gathered} 2a_{a} a_{s} f_{s} (a_{s} - (1 + \xi {}_{s})a_{a} )(a_{a} (A_{2} - A_{1} \xi_{s} ) - A_{2} ) + \xi_{s} a_{s} (f_{a} a_{s} - a_{a} f_{s} )(1 + A{}_{2})(A_{2} a_{a} - 1) + \hfill \\ a_{s} (f_{a} a_{s} - a_{a} f_{s} )(2A_{2} (a_{s} - (1 + \xi_{s} )a_{a} ) - 2a_{a} \xi_{s} (A_{2} - A_{1} \xi_{s} (1 + A_{2} )) + 2A_{2} (a_{s} (a{}_{s} - (1 + \xi_{s} )a_{a} - a_{a}^{3} \xi_{s} f_{a} ) \hfill \\ \end{gathered} }{{2A_{2} a_{a} a_{s} \theta_{a} (a_{s} - (1 + \xi_{s} )a_{a} ) - a_{a}^{2} a_{s} \theta_{a} \xi_{s} (A_{2} - A_{1} \xi_{s} )(1 + A_{2} )}} $$

So we can get the optimal profits of the platform are respectively:

$$ p_{psp}^{*} = A_{1} \frac{{A_{2} (1 + \xi {}_{s + a})(f_{a} a_{s} - f{}_{s}a_{a} )((1 + \xi {}_{s + a})(f_{s} (2a_{s} - a_{a} (A_{1} + A_{2} )) - (f{}_{a}a_{s} - f_{s} a{}_{a})) - 2a_{a} f_{s} \xi {}_{s + a})}}{{a_{s} ((1 + 2\xi {}_{s + a})(2a_{s} - a_{a} (A_{1} + A_{2} )) - 2a_{a} \xi {}_{s + a})^{2} }} $$

$$ \begin{aligned} p_{s + a}^{*} & = A_{1} \frac{\begin{gathered} A_{2} (1 + \xi {}_{s + a})(f_{a} a_{s} - f_{s} a_{a} )(\theta_{f} a_{s} (1 + 2\xi {}_{s + a})(2a_{s} - a_{a} (A_{1} + A_{2} )) \hfill \\ - 2a_{s} \theta_{f} a{}_{a}\xi {}_{s + a} + \xi {}_{s + a}((\xi {}_{s + a} + 1)a_{s} - (1 + 2\xi {}_{s + a})a_{a} )) \hfill \\ \end{gathered} }{{a_{s} ((1 + 2\xi {}_{s + a})(2a_{s} - a_{a} (A_{1}^{{}} + A_{2} )) - 2a_{a} \xi {}_{s + a})_{2} }} \\ & \quad + A_{2} \frac{\begin{gathered} ((f_{a} a{}_{s} - a_{a} f_{s} )((1 + \xi_{s + a} )((1 + \xi_{s + a} )a_{s}^{{}} - \xi_{s + a} a_{a} ) - a_{a} \xi_{s + a} )) \hfill \\ ((f_{a} a{}_{s} - a_{a} f_{s} )((1 + 2\xi_{s + a} )(1 - \xi_{s + a} - A_{1} - A_{2} )a_{a} + (1 + \xi_{s + a} )a_{s} ) - 3a_{a} \xi_{s + a} ) \hfill \\ \end{gathered} }{{a_{s} a_{a} \theta_{a} ((1 + 2\xi_{s + a} )(2a_{s} - a{}_{a}(A_{1} + A_{2} )) - 2a_{a} \xi_{s + a} )^{2} }} \\ & \quad - NW + FR - IF \\ \end{aligned} $$

The basic utility of the driver is to be satisfied $f_{i} < 2a_{i}$.

By considering heterogeneous passengers, we can see that this model is robust. We find that when there are two types of heterogeneous passengers, the pure aggregation business model platform self-operation + aggregation business model platform is compared, and passengers who are more sensitive to price and service quality are more inclined to self-operation + aggregation business model platform. Passengers who are more sensitive to price than service quality are more inclined to pure aggregation business model platform. Because although passengers who are more sensitive to price can satisfy themselves in both business models when they need to serve, choosing a platform with a pure aggregation business model can reduce the choice time cost. When choosing a self-operated d + aggregated business model platform, drivers with higher prices and service quality may be matched.

In this section, by considering heterogeneous passengers and comparing the three business models, it is found that passengers who are more sensitive to the price of service quality are more inclined to the platform related to the self-operated business model, and passengers who are more sensitive to the price of service quality are more inclined to the platform related to the aggregated business model.

The pure self-operated business model platform should improve the service quality of the platform to the driver while improving the service quality to the passengers. Of course, higher prices can also be set, because passengers who are more sensitive to the service quality are willing to pay higher prices to experience better services. In this way, the pure self-operated business model platform can expand the supply side while expanding the demand side to increase the profit of the platform; The pure aggregation business model platform should improve the quality of service for drivers while reducing the quality of service for passengers, so that a lower price can be set, because passengers who are more sensitive to prices are willing to pay lower prices to obtain poor quality services, so that the pure aggregation platform can also expand the supply side while expanding the demand side to increase platform profits; Self-operated + aggregated business model platform has the above two business models at the same time, the platform can implement price discrimination while improving the service quality of drivers, and make obvious differential pricing. Then passengers with different heterogeneity can choose different services on the platform according to their different needs, so that the platform of self-management + aggregation business model can also improve the profit of the platform.

## Conclusion and future prospects

The development of the sharing economy and the aggregation model platform has greatly promoted the development of the online car-hailing market, and has found a new online car-hailing business model, which has largely supplemented the market vacancy, while providing people with a more convenient and more choice platform for travel. In this paper, an analysis model is used to capture the optimal business model selection of the online car-hailing platform in the presence of the driver’s preference for the service platform when the large-flow platform enters the market in the online car-hailing market, and the rights and interests of the driver are also mentioned.

The research results show that: First, the joining fee received by the platform in the aggregation mode is not high. When it is impossible to make positive feedback on the cost of the platform in the self-operated mode, and there is a risk of increasing the dissatisfaction rate of the original users on the platform, resulting in the loss of consumers, the pure self-operated mode should be adopted, which can maintain the customers to the greatest extent and maximize the benefits. In the aggregation mode, the platform can ensure that the original user’s dissatisfaction rate with the platform is at a low level, in order to maintain user stickiness. When the driver is more sensitive to the heterogeneity of service quality, the business model has lower qualification requirements for the driver, which is conducive to expanding supply and making sufficient preparations for better seizing the market. Therefore, in this case, the platform should adopt a pure aggregation business model 3. Under the aggregation mode, when the consumer’s dissatisfaction rate with the platform is at a low level and the cost of the platform under the self-operated mode can be controlled within a low range, the platform should adopt the self-operated + aggregation business model, which can not only give full play to the flow advantage of the platform, realize the flow realization, expand the supply, but also maximize the profit and achieve Pareto improvement.4. When there are heterogeneous passengers, the pure self-management business model platform should improve the service quality of the platform to the driver while improving the service quality to the passengers, and of course, higher prices can be set; the pure aggregation business model platform should improve the quality of service for drivers while reducing the quality of service for passengers, so that a lower price can be set; the self-operated + aggregated business model platform can implement price discrimination while improving the quality of driver services, and make obvious differential pricing.

This study has a certain explanation and explanation for the reality. The flywheel effect of users and drivers in the current online car-hailing market shows that drivers are more inclined to serve better self-operated platforms when they have compliance qualifications, thus forming industry barriers. At present, the government’s supervision of the online car-hailing industry is continuously strengthened, and the road of compliance is imperative. Therefore, the platform should improve the service quality for drivers, so as to obtain a larger market by optimizing the supply side. The online car-hailing platform should also collect drivers’ dissatisfaction through multiple channels, such as questionnaires to collect drivers’ opinions and suggestions on working conditions, income, platform policies, etc. The driver’s satisfaction is indirectly judged by analyzing the driver’s working mode (such as the order frequency, the proportion of cancelled orders, online time, etc.). In order to effectively respond to the driver’s dissatisfaction, the online ride-hailing platform needs to establish a good communication mechanism to ensure that the driver’s voice can be heard and respond and process the feedback in a timely manner. At the same time, the platform should continuously optimize the management system based on the collected information to improve the working environment and income of drivers.

There are still many deficiencies in this study. First of all, we only study the static pricing model, and do not study the dynamic pricing; secondly, we only study the impact of the driver’s side on the platform’s profit. Since both bilateral platforms have network externalities, both passengers and drivers will have an impact on the platform’s profit; finally, we ignore the uncertainty of the external market, including the uncertainty of passenger demand, the uncertainty of driver supply, and the uncertainty of platform service quality. These will be the direction of our future research.

### Supplementary Information

Supplementary Information.

## Data Availability

Data is provided within the manuscript or supplementary information files.
