# Supplementary material for: The platform business model selection of online ride-hailing giants based on the aggregation model
Source: Sci Rep. 2024 Apr 13;14:8574. doi: 10.1038/s41598-024-58984-x (PMC11369292; doi:10.1038/s41598-024-58984-x)
Supplement: Supplementary file 1 — Supplementary Information. [file 41598_2024_58984_MOESM1_ESM.pdf]

Appendix :

Proposition 1 proof:

Proof : In order to obtain the maximum profit of the pure self-operation model. Solved $P_{psp}=\left[\beta\frac{1-\gamma}{\gamma}+(1-\beta)\frac{1-\alpha}{\alpha}\right]h_s\frac{f_s-h_s}{a_s}-NW$ first partial derivative about h_s .let $\frac{\partial P_{PSP}}{\partial h_s}=0$,The optimal reward of the driver is $h_s^*=\frac{f_s}{2}$,By solving P_{PSP} second-order partial derivative about h_s , $\frac{\partial^2 P_{PSP}}{\partial h_s^2}=-\frac{2(1-\gamma)\beta}{\gamma a_s}-\frac{(1-\gamma)(1-\beta)}{\alpha a_S}<0$,So the optimal solution of the profit model is obtained at h_s first partial derivative.Based on the driver 's rational constraints.Bring h_s^* into the driver utility model to get $0\le f_s\le 2a_s$,Let h_s^* into P_{PSP} , o_s and s_s ,obtained respectively

$$P_{psp}^*=\left[\frac{\beta(1-\gamma)}{\gamma}+\frac{(1-\alpha)(1-\beta)}{\alpha}\right]\frac{f_s^2}{4a_s}-NW,\quad o_s^*=\frac{f_s}{2\gamma}+\frac{f_s}{2\alpha},\quad s_s^*=\frac{f_s}{2a_s}$$

According to the optimal result, we find P_{PSP}^* first-order partial derivative about a_s ,and $\frac{\partial P_{PSP}^*}{\partial a_s}=-\frac{\beta(1-\gamma)(f_s-c_s)^2}{4\gamma a_s}-\frac{(1-\beta)(1-\gamma)(f_s-c_s)^2}{4\alpha a_s}<0$,Then find s_s^* first-order partial derivative about a_s ,and $\frac{\partial s_s^*}{a_s}=-\frac{f_s-c_s}{2a_s^2}<0$. find P_{PSP}^* first-order partial derivative about f_s , $\frac{\partial P_{PSP}^*}{\partial f_s}=\frac{\beta(1-\alpha)(f_s-c_s)}{2\gamma a_s}+\frac{(1-\beta)(1-\alpha)(f_s-c_s)}{2\alpha a_s}>0$ .At last,find P_{PSP}^* first-order partial derivative about β ,and $\frac{\partial P_{PSP}^*}{\partial\beta}=\frac{(1-\gamma)(f_s-c_s)^2}{4\gamma a_s}-\frac{(1-\alpha)(f_s-c_s)^2}{4\alpha a_s}$,when $\alpha>\gamma,\frac{\partial_{PSP}^*}{\partial\beta}>0$ .when $\alpha<\gamma,\frac{\partial_{PSP}^*}{\partial\beta}<0$.

According to formula (1), without considering other variables. u_S increases monotonously with f_s .

Proposition 2 proves :

It is proved that in order to solve the maximum profit of the platform under the pure aggregation business model, the solution of the $P_{PAP}=\mu\frac{1-\alpha}{\alpha}h_a\left(\frac{f_a-h_a}{a_a}\varphi+\frac{f_a-h_a}{a_a}(1-\varphi)\right)+IF-FR$ about h_a first-order partial derivative,let $\frac{\partial P_{PAP}}{\partial h_a}=0$,get the optimal reward for the driver $h_a^*=\frac{f_a}{2}$.

Because $\frac{\partial^2 P_{PAP}}{\partial h_a^2}=-\frac{2\mu(1-\alpha)}{\alpha a_a}<0$,Therefore, the maximum profit P_{PAP}^* of the platform is obtained at h_a^* .Based on the driver 's rational constraints,let h_a^* into the driver 's utility model to get $0\le f_a\le 2a_a$ .let h^* into P_{PAP} , o_a and s_a ,obtained respectively $s_a^*=\frac{f_a}{2a_a}$, $o_a^*=\frac{f_a}{2\alpha}$ and

$$P_{PAP}^*=\frac{\mu(1-\alpha)f_a^2}{4\alpha a_a}+IF-FR\cdot$$

For the optimal results, we calculate the first-order partial derivative of P_{PAP}^* with respect to a_a ,and $\frac{\partial P_{PAP}^*}{\partial a_a}=-\frac{\mu(1-\alpha)f_a^2}{4\alpha a_a}<0$ .Then the first-order partial derivative of s_a^* with respect to a_a is

obtained,and $\frac{\partial s_a^*}{\partial a_a}=-\frac{f_a}{2a_a}<0$ .At last the first-order partial derivative of P_{PAP}^* with respect to f_a is obtained,and $\frac{\partial P_{PAP}^*}{\partial f_a}=\frac{\mu(1-\alpha)f_a}{2\alpha a_a}>0$.

Proposition 3 Proof

Proof : In order to facilitate calculation,let $\theta_a=a_a-a_s$,$\theta_f=f_a-f_s$,$k_1=\frac{\beta(1-\gamma)}{\gamma}\frac{(1-\alpha)(1-\beta)}{\alpha}$, $k_2=\frac{\mu(1-\alpha)}{\alpha}$ .In order to solve the optimal results of the online car-hailing platform under the self-operated + aggregated business model, the first-order partial derivatives of P_{s+a} with respect to h_{+a} and h_{+s} are solved respectively.let $\frac{\partial P_{s+a}}{\partial h_{+a}}=0$ and $\frac{\partial P_{s+a}}{\partial h_{+s}}=0$,and obtained $h_{+a}^*=\frac{(k_1^2-k_1k_2)a_a\theta_f+2k_1k_2f_a\theta_a}{4k_1k_2a_s-(k_1+k_2)^2a_a}$ and $h_{+s}^*=\frac{(k_1^2+k_1k_2)(f_a\theta_a-a_a\theta_f)+2k_1k_2\theta_f a_s}{4k_1k_2a_s-(k_1+k_2)^2a_a}$.

Then the second-order partial derivatives of P_{s+a} with respect to h_{+a} and h_{+s} and the second-order mixed partial derivatives of P_{s+a} with respect to h_{+a} and h_{+s} are solved respectively.,obtained $\frac{\partial^2 P_{s+a}}{\partial h_{+s}^2}=-\frac{2k_1}{\theta_a}<0$, $\frac{\partial^2 P_{s+a}}{\partial h_{+a}^2}=-\frac{2k_2a_s}{a_a\theta_a}<0$ and $\frac{\partial P_{s+a}}{\partial h_{+a}\partial h_{+s}}=\frac{k_1+k_2}{\theta_a}$ .In order to prove the optimality of the first derivative, the Hessian matrix is introduced. When the Hessian matrix is a positive definite matrix, there is a minimum value ; when the Hessian matrix is a negative definite matrix, there is a maximum value, so the Hessian matrix should be negative. The Hessian matrix under the second derivative condition is $\begin{bmatrix}-\frac{2k_2a_a}{a_s\theta_a} & \frac{k_1+k_2}{\theta_a}\\ \frac{k_1+k_2}{\theta_a} & -\frac{2k_1}{\theta_a}\end{bmatrix}$ .Therefore, the odd-order principal subformula of the matrix is negative, and the even-order principal subformula is positive. It is known that $-\frac{2k_2a_a}{a_s\theta_a}<0$,so $\begin{vmatrix}-\frac{2k_2a_a}{a_s\theta_a} & \frac{k_1+k_2}{\theta_a}\\ \frac{k_1+k_2}{\theta_a} & -\frac{2k_1}{\theta_a}\end{vmatrix}>0$,and then obtained $\frac{a_s}{a_a}<\frac{4k_1k_2}{(k_1+k_2)^2}$.

According to the rational constraints of the individual driver, when ensuring non-negative supply, it is necessary to make : $u_{+a}=0$、 $u_{+a}=u_{+s}$ .At this time, two indifference points can be obtained : $\omega_{+1}=\frac{f_s-h_{+s}-f_a-h_{+a}}{a_s-a_a}$、 $\omega_{+2}=\frac{f_a-h_{+a}}{a_a}$.

To make the above h_{+a}^*、 h_{+s}^*、 θ_a、 θ_c、 θ_f constraint holds,the following constraints need to be met : $\begin{cases}\omega_{+2}>0\\ \omega_{+1}-\omega_{+2}<0\\ \omega_{+1}<1\end{cases}$ .Bring h_{+a}^*、 h_{+s}^*、 θ_a、 θ_c、 θ_f into the above inequality constraint group, the following constraint conditions are obtained :

i $\frac{a_s}{a_a} < \frac{4k_1k_2}{(k_1+k_2)^2}$ or $\frac{a_s}{a_a} \le \frac{k_2}{k_1}$

ii $\left[2k_1k_2a_s - \left(k_1k_2 + k_2^2\right)a_a\right]f_a - \left(k_1^2 - k_1k_2\right)a_af_s + (k_1+k_2)^2a_a^2 > 4k_1k_2a_aa_s$

In summary, the constraints of the heterogeneity ratio of driver service preference are as follows :

i $\frac{a_s}{a_a} < \frac{4k_1k_2}{(k_1+k_2)^2}$ or $\frac{a_s}{a_a} \le \frac{k_2}{k_1}$

$\left[2k_1k_2a_s - \left(k_1k_2 + k_2^2\right)a_a\right]f_a - \left(k_1^2 - k_1k_2\right)a_af_s + (k_1+k_2)^2a_a^2 > 4k_1k_2a_aa_s$

At last let h_{+a}^* and h_{+s}^* into s_{+a} , s_{+s} and P_{s+a} ,obtained respectively

$s_{+a}^* = \frac{k_1(k_1+k_2)a_af_s - 2k_1k_2a_sf_a}{a_a\left[(k_1+k_2)^2a_a - 4k_1k_2a_s\right]}$, $s_{+s}^* = \frac{2k_1k_2\theta_f - \left(k_1k_2 - k_2^2\right)f_a}{4k_1k_2a_s - (k_1+k_2)^2a_a}$ and

$$
\begin{aligned}
P_{s+a}^* &= (k_1+k_2)h_{+s}^*s_{+s}^* + (k_3+k_4)h_{+a}^*s_{+a}^* + IF_{+a} - NW_{+s} - FR_{+a} \\
&= \frac{\begin{gathered}\left(7k_1^3k_2^2 + 2k_1^2k_2^3 - 2k_1^4k_2 + k_1k_2^4\right)f_af_sa_a^2a_s + \left(18k_1^2k_2^3 + 3k_1k_2^4 + 3k_1^3k_2^2\right)f_a^2a_a^2a_s \\ + \left(2k_1^3k_2^2 - 2k_1k_2^4\right)f_a^2a_s^2a_a + \left(18k_1^4k_2 - 9k_1^3k_2^2 - 2k_1^2k_2^3 - k_1k_2^4\right)f_af_sa_a^3 + \left(k_1^4k_2 - 5k_1^2k_2^3\right)f_s^2a_a^2a_s \\ + \left(2k_1^4k_2 - k_1k_2^4 + 5k_1^3k_2^2 + 2k_1^2k_2^3\right)f_a^2a_a^3 - 4k_1^3k_2^2f_af_sa_s^2a_a + \left(k_1^4k_2 - k_1^2k_2^3\right)f_s^2a_a^3 \\ + \left(2k_1^3k_2^2 + 6k_1^2k_2^3\right)f_af_sa_s^3 + 4k_1^2k_2^3f_a^2a_s^3 + 4k_1^3k_2^2f_s^2a_s^2a_a\end{gathered}}{a_a\theta_a\left[4k_1k_2a_s - (k_1+k_2)^2a_a\right]^2} \\
&\quad - NW_{+s} + IF_{+a} - FR_{+a}
\end{aligned}
$$

Proposition 4 proves :

Proof : According to proposition 1-3, the optimal profit of the platform under each business model can be obtained :

$$P_{psp}^* = \left[\frac{\beta(1-\gamma)}{\gamma} + \frac{(1-\alpha)(1-\beta)}{\alpha}\right]\frac{f_s^2}{4a_s} - NW$$

$$P_{PAP}^* = \frac{\mu(1-\alpha)f_a^2}{4\alpha a_a} + IF - FR$$

$$
\begin{aligned}
P_{s+a}^* &= k_1h_{+s}^*s_{+s}^* + k_2h_{+a}^*s_{+a}^* + IF_{+a} - NW_{+s} - FR_{+a} \\
&= \frac{\begin{gathered}\left(7k_1^3k_2^2 + 2k_1^2k_2^3 - 2k_1^4k_2 + k_1k_2^4\right)f_af_sa_a^2a_s + \left(18k_1^2k_2^3 + 3k_1k_2^4 + 3k_1^3k_2^2\right)f_a^2a_a^2a_s \\ + \left(2k_1^3k_2^2 - 2k_1k_2^4\right)f_a^2a_s^2a_a + \left(18k_1^4k_2 - 9k_1^3k_2^2 - 2k_1^2k_2^3 - k_1k_2^4\right)f_af_sa_a^3 + \left(k_1^4k_2 - 5k_1^2k_2^3\right)f_s^2a_a^2a_s \\ + \left(2k_1^4k_2 - k_1k_2^4 + 5k_1^3k_2^2 + 2k_1^2k_2^3\right)f_a^2a_a^3 - 4k_1^3k_2^2f_af_sa_s^2a_a + \left(k_1^4k_2 - k_1^2k_2^3\right)f_s^2a_a^3 \\ + \left(2k_1^3k_2^2 + 6k_1^2k_2^3\right)f_af_sa_s^3 + 4k_1^2k_2^3f_a^2a_s^3 + 4k_1^3k_2^2f_s^2a_s^2a_a\end{gathered}}{a_a\theta_a\left[4k_1k_2a_s - (k_1+k_2)^2a_a\right]^2} \\
&\quad - NW_{+s} + IF_{+a} - FR_{+a}
\end{aligned}
$$

In order to obtain the optimal business model of the platform, the optimal profits under different business models will be compared separately. In order to facilitate the calculation, this paper sets

$$\left(7k_1^3k_2^2+2k_1^2k_2^3-2k_1^4k_2+k_1k_2^4\right)f_af_sa_a^2a_s+\left(18k_1^2k_2^3+3k_1k_2^4+3k_1^3k_2^2\right)f_a^2a_a^2a_s+\left(2k_1^3k_2^2-2k_1k_2^4\right)f_a^2a_s^2a_a+$$
$$\left(18k_1^4k_2-9k_1^3k_2^2-2k_1^2k_2^3-k_1k_2^4\right)f_af_sa_a^3+\left(k_1^4k_2-5k_1^2k_2^3\right)f_s^2a_a^2a_s+\left(2k_1^4k_2-k_1k_2^4+5k_1^3k_2^2+2k_1^2k_2^3\right)f_a^2a_a^3-4k_1^3k_2^2f_af_sa_s^2a_a$$
$$+\left(k_1^4k_2-k_1^2k_2^3\right)f_s^2a_a^3+\left(2k_1^3k_2^2+6k_1^2k_2^3\right)f_af_sa_s^3+4k_1^2k_2^3f_a^2a_s^3+4k_1^3k_2^2f_s^2a_s^2a_a=A,$$
$$a_a\theta_a\left[4k_1k_2a_s-(k_1+k_2)^2a_a\right]^2=B$$

so $P_{s+a}^*=\dfrac{A}{B}+NW_{+s}-NIF_{+a}+NFR_{+a}$ (20)

By comparing the benefits of a P_{psp}^* and P_{pap}^*, we get

$$p_{psp}^*-P_{pap}^*=\frac{a_ak_1f_s^2-a_sk_2f_a^2}{4a_aa_s}+NFR-NW-NIF \tag{21}$$

comparing the benefits of a P_{psp}^* and P_{s+a}^*, we get

$$P_{psp}^*-P_{s+a}^*=\frac{k_1Bf_s^2-4Aa_s}{4Ba_s}-NIF_{+a}+NFR_{+a} \tag{22}$$

comparing the benefits of a P_{pap}^* and P_{s+a}^*, we get

$$P_{pap}^*-P_{s+a}^*=\frac{k_2Bf_a^2-4Aa_a}{4Ba_a}+NW_{+s} \tag{23}$$

When Eq. (21) and Eq. (22) are positive at the same time, the pure self-operation mode should be selected.let $\frac{a_ak_1f_s^2-a_sk_2f_a^2}{4a_aa_s}+NFR-NW-NIF>0$,we can obtained

$W_{+s}+IF_{+a}<\frac{k_1f_s^2a_a-k_2f_a^2a_s}{N4a_aa_s}+FR_{+a}$ let $\frac{k_1Bf_s^2-4Aa_s}{4Ba_s}-NIF_{+a}+NFR_{+a}>0$,we can obtained

$IF_{+a}<\frac{k_1Bf_s^2-4Aa_s}{N4Ba_s}+FR_{+a}$ .If the pure polymerization business model is selected as the optimal business model, the required type (21)is negative and the type (23) is positive. At this time can be obtained

$FR_{+a}<\dfrac{k_2f_a^2a_s-k_1f_s^2a_a}{N4a_aa_s}+IF_{+a}+W_{+s}$

$$\left[k_1k_2\left(k_1^2-k_2^2\right)\theta_ff_a+2k_1^2k_2(k_1-k_2)\theta_f^2-2k_1k_2^2(k_1+k_2)f_a(f_a+f_s)-4k_1^2k_2^2f_a\theta_f\right]a_aa_s+$$
$$\left[2k_1k_2^2(k_1+k_2)f_af_s-k_1k_2\left(k_1^2-k_2^2\right)\theta_ff_s\right]\frac{a_a}{a_s}+\left[2k_1k_2^2(k_1+k_2)f_a^2+4k_1^2k_2^2f_a\theta_f\right]\frac{a_s}{a_a}<0$$

If the self-management + aggregation business model is selected as the optimal business model, then the demand type(22)and(23) are negative. At this time can be obtained

$FR_{+a}<\dfrac{4Aa_s-k_1Bf_s^2}{N4Ba_s}+IF_{+a}$， $W_{+s}<\dfrac{4Aa_a-k_2Bf_a^2}{N4Ba_a}$.

$$\varepsilon:\left[k_1k_2\left(k_1^2-k_2^2\right)\theta_ff_a+2k_1^2k_2(k_1-k_2)\theta_f^2-2k_1k_2^2(k_1+k_2)f_a(f_a+f_s)-4k_1^2k_2^2f_a\theta_f\right]a_aa_s+$$
$$\left[2k_1k_2^2(k_1+k_2)f_af_s-k_1k_2\left(k_1^2-k_2^2\right)\theta_ff_s\right]\frac{a_a}{a_s}+\left[2k_1k_2^2(k_1+k_2)f_a^2+4k_1^2k_2^2f_a\theta_f\right]\frac{a_s}{a_a}<0$$

Then we define the threshold $\overline{W_{+s}+IF_{+a}}=\frac{k_1f_s^2a_a-k_2f_a^2a_s}{N4a_aa_s}+FR_{+s}$ ， $\overline{IF_{+a}}=\frac{k_1Bf_s^2-4Aa_s}{N4Ba_s}+FR_{+a}$ ，

$\overline{FR_{+a}} = \frac{k_2 f_a^2 a_s - k_1 f_s^2 a_a}{N4a_a a_s} + IF_{+a} + W_{+s}$,so $\overline{FR_{+a}}' = \frac{4Aa_s - k_1 Bf_s^2}{N4Ba_s} + IF_{+a}$, $\overline{W_{+s}} = \frac{4Aa_a - k_2 Bf_a^2}{N4Ba_a}$,In summary, proposition 4 can be obtained.
